# Baicalin alleviates mastitis in dairy cows by targeting IL-17RA to inhibit IL-17 signaling pathway activation

**DOI:** 10.1186/s40104-026-01401-2

**Published:** 2026-04-16

**Authors:** Rui Feng, Hefei Huang, Qian Ma, Weilin Gao, Xu Chen, Xiaoxue Yan, Fan Wang, Qian Zhang, Yu Cao, Han Zhang, Junyang Teng, Xin Ran, Yong Zhang, Shoupeng Fu, Jun Liu, Xu Liu

**Affiliations:** 1 Key Laboratory of Animal Biotechnology of the Ministry of Agriculture, College of Veterinary Medicine, Northwest Agriculture & Forestry University, Yangling, Shaanxi 712100 China; 2 State Key Laboratory for Diagnosis and Treatment of Severe Zoonotic Infectious Diseases, Key Laboratory for Zoonosis Research of the Ministry of Education, Institute of Zoonosis, College of Veterinary Medicine, Jilin University, Changchun, 130062 China

**Keywords:** Baicalin, IL-17RA, IL-17 signaling pathway, Mastitis, TNF signaling pathway

## Abstract

**Background:**

Mastitis, one of the most prevalent inflammatory diseases in female mammals, causes significant economic losses in livestock farming. Notably, the natural flavonoid compound baicalin exhibits potent anti-inflammatory activity. However, its efficacy in alleviating mastitis severity and the underlying molecular mechanisms remain unexplored. Therefore, this study aims to investigate the protective effects of baicalin in alleviating mastitis and its key molecular mechanisms.

**Results:**

This study demonstrated in vivo that baicalin effectively alleviates mastitis symptoms in dairy cows and mice, primarily manifested by reduced tissue pathological damage, decreased levels of pro-inflammatory cytokines, and maintain the integrity of the blood-milk barrier (BMB). Multi-omics sequencing analysis indicated that IL-17 and TNF signaling pathways play crucial roles in this process. Further studies demonstrated that *IL-17RA*^−/−^ mice exhibited a phenotype similar to that observed with baicalin treatment, confirming the importance of this pathway. Notably, network pharmacology screening combined with molecular dynamics simulations revealed stable binding of baicalin to IL-17RA, suggesting that baicalin exerts its protective effect to alleviate mastitis by targeting IL-17RA. Mechanistically, both baicalin treatment and IL-17RA deletion block activation of key downstream pathways of the IL-17 signaling pathway, including MAPK, ERK and NF-κB, thereby suppressing excessive activation of the TNF signaling pathway, preventing exacerbation of the inflammatory response and barrier damage.

**Conclusions:**

In conclusion, this study demonstrates that baicalin inhibits excessive activation of the IL-17/TNF signaling pathway by targeting IL-17RA, thereby reducing inflammatory responses and BMB damage within the mammary gland and alleviating mastitis severity.

**Supplementary Information:**

The online version contains supplementary material available at 10.1186/s40104-026-01401-2.

## Introduction

Mastitis, a common disease primarily caused by bacterial infection, is widespread among dairy animals, resulting in significant economic losses while also posing a major public health risk to humans [1, 2]. This disease not only directly reduces milk yield and deteriorates milk quality, but can also cause irreversible damage to mammary gland tissue in severe cases and even trigger systemic inflammatory responses, thereby threatening both animal welfare and human health [3, 4]. A key determinant of mastitis pathogenesis and clinical prognosis is the structural and functional integrity of the blood-milk barrier (BMB) [5]. This specialized physiological structure, formed by tight junctions (TJ) between mammary epithelial cells, selectively regulates the transport of blood components and pathogens into milk [6]. During mastitis progression, BMB dysfunction is recognized as a critical marker of disease progression. It facilitates massive infiltration of inflammatory mediators and immune cells into mammary tissue while causing retrograde leakage of milk components into the bloodstream and exacerbates local inflammation and may trigger systemic inflammatory responses [4, 7]. Therefore, elucidating the key regulatory mechanisms governing the shift from controlled immune defense to uncontrolled hyperinflammation and BMB damage during mastitis holds significant scientific and clinical value.

In autoimmune and inflammatory diseases, diverse tissue cells and their secreted cytokines are core participants in the host's defense against pathogen invasion [8, 9]. In mastitis, following pathogen invasion, mammary epithelial cells rapidly initiate innate immune responses, while other immune and non-immune cells within the mammary gland actively participate in this process, collectively coordinating the body's rapid immune response, a biological process involves the precise regulation of multiple chemokines and cytokines [10–12]. Recent studies indicate that the interleukin-17 (IL-17) cytokine family plays a pivotal role in the interaction between the immune system and epithelial tissues, especially occupying a central position in initiating the host's anti-pathogen immune mechanisms [13, 14]. Importantly, IL-17A exerts significant regulatory functions in the host response to bacterial infections through IL-17RA-mediated signal transduction [15, 16]. Furthermore, multiple studies in bovine and murine mastitis models have observed significantly elevated expression levels of IL-17A and tumor necrosis factor α (TNFα) in mammary tissue and milk, suggesting their critical involvement in the pathological progression of mastitis [17, 18]. However, the precise role of IL-17RA signaling transduction in mastitis progression, its mechanism of action in BMB destruction, and its potential molecular networks regulating the inflammatory cascade within mammary gland remain incompletely elucidated. Therefore, investigating the role of the IL-17RA-mediated IL-17 signaling pathway in mastitis is crucial for deepening our understanding of its pathogenesis and developing novel therapeutic strategies.

Baicalin, a natural flavonoid compound, exhibits significant biological properties including potent antioxidant and anti-inflammatory effects [19, 20]. Recent studies demonstrate that baicalin effectively alleviates pathological progression and regulates immune homeostasis in various inflammation-related diseases, including lung injury, pancreatitis, enteritis, peritonitis, periodontitis, and arthritis [21–26]. Notably, baicalin has been demonstrated to inhibit inflammation mediated by the IL-17 signaling pathway [23]. Although studies have reported its efficacy in alleviating mastitis symptoms, the precise molecular mechanism remains unclear; in particular, whether it acts by regulating with the IL-17 signaling pathway requires experimental validation [27, 28]. Therefore, systematically elucidating the regulatory relationship between baicalin and the IL-17 signaling pathway holds significant value for revealing its therapeutic potential in mastitis and for the development and application of antibiotic alternatives.

This study aimed to elucidate the protective mechanism by which baicalin alleviates mastitis and to evaluate the pivotal role of the IL-17/TNF signaling pathway as an intramammary “inflammatory amplifier”. Therefore, this study employed dairy cow and *IL-17RA* gene knockout (KO) mice models to reveal novel insights into the molecular mechanisms underlying baicalin's therapeutic effect, validate the therapeutic potential of targeting IL-17RA, and assess the feasibility of developing baicalin as an antibiotic alternative or adjunctive therapy.

## Materials and methods

### Ethics statement

Wild-type and *IL-17RA*^−/−^ mice were housed in specific pathogen-free facilities at the Experimental Animal Center of Northwest Agriculture & Forestry University. The *IL-17RA*^−/−^ mice were purchased from Cyagen (Suzhou, China). Dairy cows used in this study were raised and experimentally handled at the animal base. All animal procedures were approved by the Northwest Agriculture & Forestry University Animal Ethics Committee (Approval No. IACUC2024-1108 and NWAFU-20220256) and conducted in strictly adhered to the “Guidelines for Animal Care and Use for Research Purposes”.

### Cell culture and treatment

 Mouse mammary epithelial cell line (EPH4, C2180; kindly provided by Shanghai WheLab) and MAC-T cells (stored in this laboratory) were cultured as previously described [1, 2]. The culture medium consisted of 90% Gibco DF12 medium, 9% Gibco fetal bovine serum, and 1% penicillin–streptomycin. All cell experiments were performed under sterile conditions to prevent contamination. An in vitro mastitis model was established by treating cells with 5 μg/mL LPS (Sigma, USA) for 12 h. Baicalin (purity ≥ 95%) was purchased from Macklin (Shanghai, China), with a concentration of 20 μmol/L for 24 h. Furthermore, the TNFα inhibitor SPD304 and recombinant IL-17A (rIL-17A) were purchased from MedChemExpress (MCE, USA) and were used at concentrations of 5 μmol/L (for 2 h) and 100 ng/mL (for 12 h), respectively.

### Mouse mastitis model

A mouse mastitis model was established based on previously reported methods [1, 2]. Briefly, lactating female mice with the same genetic background (within 14 d postpartum, body weight 20–23 g, 3 mice per group) were used. A suspension of *Escherichia coli* (*E. coli*) (CVCC1418; 1 × 10^7^ CFU in 50 μL) was injected into the fourth and fifth pairs of mammary glands. After 24 h of infection under standard housing conditions, the mice were euthanized, and mammary tissues were collected for further analysis.

To evaluate the therapeutic effect of baicalin, mice were orally administered 200 mg/kg baicalin daily for three weeks. In the third week (first week postpartum), the mastitis model was established on d 6 as described above, and samples were collected.

### Dairy cow mastitis model

The dairy cow mastitis model was established following previously described procedures [1, 2]. Holstein cows with similar genetic backgrounds (average body weight 590 ± 15.30 kg) were divided into three groups (three cows per group): control, mastitis, and baicalin treatment. The control group received a standard diet; the mastitis group was fed a standard diet and infected with *E. coli* (ATCC25922; 6 × 10^6^ CFU/mL) for 3 d before sampling; the baicalin group received a diet supplemented with baicalin and was similarly infected. The experiment lasted five weeks, including one week of acclimatization. Baicalin was administered twice daily (7:00 and 17:00) at 25 g per feeding. Cows were housed individually in clean pens with free access to feed and water, and milking was performed daily. Mastitis tests were conducted three times per week. Mammary tissues were collected after the trial for subsequent analyses.

### Somatic cell count (SCC)

SCC experiments were conducted according to previously reported methods [2], with measurements performed using the Countess™ 3 automated cell counter (AMQAX2000, Thermo Fisher Scientific, USA).

### CCK-8 assay

Cell viability was assessed using a CCK-8 kit (96992, Sigma, USA). In brief, 10 μL of CCK-8 reagent was added to each well, and absorbance was measured at 450 nm using a microplate reader.

### RT-qPCR

RT-qPCR was performed according to previously reported methods [2, 29]. Briefly, total RNA was extracted from cells or mammary tissues using TRIzol reagent (15596026CN, Thermo Fisher Scientific, USA) and reverse-transcribed into cDNA using a reverse transcription kit (Takara Bio Inc., China). Quantitative PCR was performed using 0.3 μL each of forward and reverse primers, 5 μL FastStart Universal SYBR Master (Roche, USA), and 3.4 μL RNase-free water in a total reaction volume of 10 μL. Gene expression was quantified using QuantStudio Design and Analysis Software (Applied Biosystems, USA). Primer sequences are listed in Tables S1 and S2.

### Western blotting

Antibody information is provided in Table S3. This experiment was performed according to previously reported methods [2, 29]. Briefly, cells or tissues were lysed in RIPA buffer containing protease inhibitors (Santa Cruz, USA), and protein concentrations were determined using an Enhanced BCA Protein Assay Kit (P0009, Beyotime, China). Proteins were separated by SDS-PAGE on 10% or 12% gels, transferred to PVDF membranes (Roche, USA), and incubated with primary and secondary antibodies. Protein bands were visualized using an ECL detection system (ChemiDoc Image Analysis System, BIO-RAD, USA) and analyzed with ImageJ software (NIH, Bethesda, MD, USA).

### Immunofluorescence (IF)

IF was performed to examine the expression and distribution of TJ proteins in cells and mammary gland tissues, following previously reported methods [2, 29]. Mammary gland tissue sections were prepared by Shaanxi Yike Biological Technology Co., Ltd. (Shaanxi, China), and images were captured using a fluorescence microscope (Nikon, Tokyo, Japan). For cells, samples were fixed, permeabilized, and incubated with primary and secondary antibodies. Nuclei were stained with DAPI (C1002, Beyotime, China), and images were acquired using a fluorescence microscope (Nikon, Tokyo, Japan). Antibody details are listed in Table S3.

### Hematoxylin-eosin (HE) staining

Mammary tissues were fixed in 4% paraformaldehyde and sent to Shaanxi Yike Biological Technology Co., Ltd. (Shaanxi, China) for HE staining. Stained sections were examined under an optical microscope (Olympus, Japan).

### Transmission electron microscope

Transmission electron microscope was performed according to previously reported method [2]. Briefly, mammary tissues were fixed and processed by Chengdu Lilai Biotechnology Co., Ltd. (Chengdu, China). First, samples underwent pre-fixation with 2.5% glutaraldehyde, followed by re-fixation with 1% osmic acid. After acetone gradient dehydration (30%, 50%, 70%, 80%, 90%, 95%, and 100% ethanol, with the 100% step repeated three times), samples were treated with a mixture of dehydrating agent and embedding agent at a ratio of 3:1, 1:1 and 1:3, respectively, and then embedded in pure resin. Subsequently, ultrathin Sects. (60–90 nm) were prepared and mounted on copper grids. These sections underwent uranium acetate staining for 10–15 min followed by lead citrate staining at room temperature for 1–2 min. Finally, observations and imaging were performed using a JEM-1400FLASH transmission electron microscope (JEOL, Japan).

### *IL-17RA*-KO cell line

Briefly, first, obtain the CDS sequence information for mouse IL-17RA from the NCBI website (Table S4). Subsequently, analyze and select highly-scoring sgRNAs based on this sequence from the CRISPOR website (http://crispor.tefor.net/), relevant information is listed in Table S5. Next, evaluate the cleavage activity of the sgRNAs using the previously reported SSA method [1], construct Cas9 (PX458, Addgene) targeting vectors with the selected sgRNAs and electroporate the targeting vectors into EPH4 following established protocols [2]. Finally, identify positive *IL-17RA*-KO EPH4 based on Sanger sequencing results and expand the cultures for subsequent experiments.

### Computational processing and bioinformatics of RNA-seq

The samples from different treatment groups were collected and sent to Novogene Co., Ltd. (Beijing, China) for RNA-seq. Reference genomes for mice were obtained from the Ensembl website (https://asia.ensembl.org/index.html). Data were deposited in the NCBI BioProject database (https://www.ncbi.nlm.nih.gov/sra/). Differentially expressed genes were identified with a false discovery rate of less than 0.05 and |log_2_Fold Change| > 1. Data analysis was performed using the Novogene Cloud Platform (https://magic.novogene.com/customer/main#/homeNew).

### Computational processing and bioinformatics of proteomics

The samples from different treatment groups were collected and sent to Novogene Co., Ltd. (Beijing, China). The cow reference genome was obtained from Ensembl website (https://asia.ensembl.org/index.html). The proteomic data had been deposited in the Proteomics Identification Database (https://www.ebi.ac.uk/pride/). Differentially expressed proteins were identified with a significance threshold of *P* < 0.05. Data were analyzed using the Novogene Cloud Platform (https://magic.novogene.com/customer/main#/homeNew).

### Molecular dynamics (MD) simulation

MD simulations were performed using Gromacs 2022 following previously reported protocols [2]. Following the simulations, the binding free energies between the IL-17RA protein and baicalin ligand were calculated using the g_mmpbsa program based on the MM/PBSA methodology. This approach decomposes the total binding free energy into contributions from van der Waals, electrostatic, and solvation energies, providing a quantitative measure of the interaction affinity and stability. Subsequently, the simulated trajectories were visualized and analyzed using VMD software to evaluate conformation stability and flexibility by calculating metrics such as root mean square deviation and root mean square fluctuation. Moreover, PyMOL was employed to generate high-quality three-dimensional structural images, clearly illustrating key binding interactions such as hydrogen bonds and hydrophobic contacts at the binding site, thereby facilitating detailed interpretation and preparation of publication-quality figures.

### ELISA

ELISA was performed according to the manufacturer’s instructions. Bovine IL-6 (F4043-A), IL-1β (F4049-A) and TNFα (F6720-B) kits were purchased from FANKEW (Shanghai, China). Mouse IL-6 (JL20268), IL-1β (JL18442) and TNFα (JL10484) kits were obtained from Jianglai Biological (Shanghai, China).

### Virtual screening of small molecules targeting the IL-17RA protein

This analysis was conducted by Chengdu Tianji Computing Technology Co., Ltd. (Chengdu, China). Briefly, the IL-17RA protein sequence was obtained from the Uniprot database. First, its structure was simulated using AlphaFold3, followed by energy optimization with Rosetta Relax. The obtained 3D protein structure underwent dehydration and hydrogenation using PyMOL software. Subsequently, to further approximate the IL-17RA protein's true structure, a 10 ns molecular dynamics simulation was performed using Gromacs 2023.3 to converge the protein structure. Small molecules were then screened based on the TCMBANK compound library. The obtained SMILES molecular formats were analyzed using RDKit to eliminate erroneous small molecule information, such as incorrect bond entries (preliminary screening). This yielded a valid screening library of 29,365 small molecules for virtual screening. Finally, high-throughput virtual screening was performed using Autodock Vina 1.2.3. The relevant screening results were shown in Table S6.

### Statistical analysis

Data are presented as mean ± SEM from at least three independent experiments. Statistical analysis was performed using SPSS 20 and GraphPad Prism 8.0. Unpaired *t*-tests were used for comparisons between two groups, and one-way ANOVA followed by Tukey’s post-hoc test was applied for multiple comparisons. Significance levels are indicated as follows: ^*^P < 0.05, ^**^P < 0.01, and ns represents *P* > 0.05.

## Results

### Activation of the IL-17 signaling pathway exacerbates EPH4 inflammatory responses and TJ damage

To validate the importance of IL-17 signaling pathway activation in mastitis progression, an *IL-17RA*-KO EPH4 cell model was established, and an in vitro mastitis cell model was created using LPS and rIL-17A for preliminary validation.

**Fig. 1:**
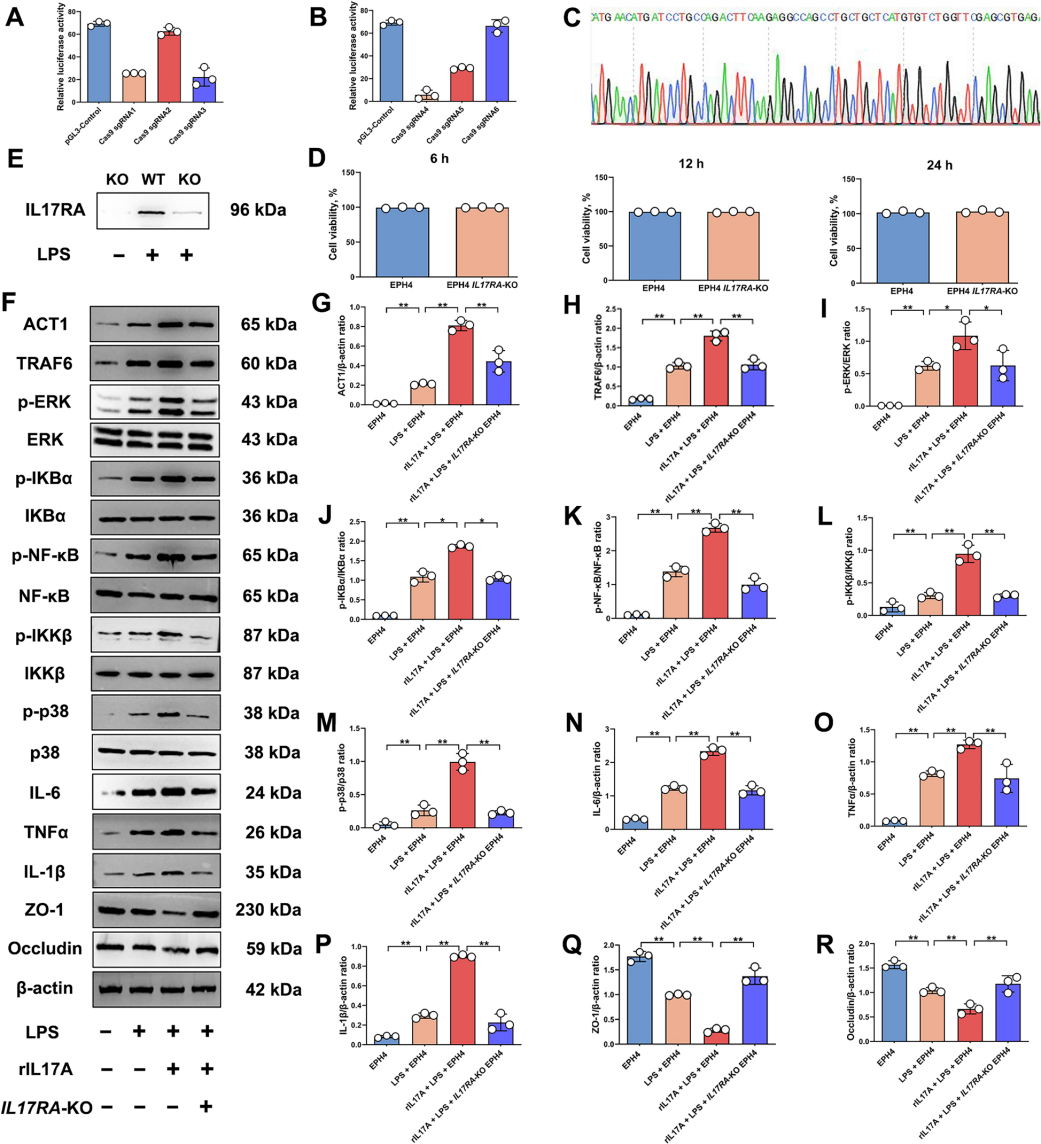
IL-17RA deletion alleviates EPH4 inflammatory responses and TJ damage. **A** and **B** Analysis of sgRNA cleavage activity. **C** Sanger sequencing identification of *IL-17RA*-KO EPH4. **D** CCK-8 assay for cell viability in EPH4 and *IL-17RA*-KO EPH4 cultured for 6, 12 and 24 h. **E** Detection of IL-17RA protein expression levels. **F** Detection of ACT1, TRAF6, p-ERK, ERK, p-IKBα, IKBα, p-NF-κB, NF-κB, p-IKKβ, p-IKKβ, p-p38β, p38, IL-6, IL-1β, TNFα, ZO-1 and Occludin protein expression levels. **G** Analysis of ACT1 and β-actin protein expression levels. **H** Analysis of TRAF6 and β-actin protein expression levels. **I** Analysis of p-ERK and ERK protein expression levels. **J** Analysis of p-IKBα and IKBα protein expression levels. **K** Analysis of p-NF-κB and NF-κB protein expression levels. **L** Analysis of p-IKKβ and IKKβ protein expression levels. **M** Analysis of p-p38 and p38 protein expression levels. **N** Analysis of IL-6 and β-actin protein expression levels. **O** Analysis of TNFα and β-actin protein expression levels. **P** Analysis of IL-1β and β-actin protein expression levels. **Q** Analysis of ZO-1 and β-actin protein expression levels. **R** Analysis of Occludin and β-actin protein expression levels. Values are expressed as mean ± SEM (*n* = 3 per group), * indicates significant difference (*P* < 0.05), ** indicates highly significant difference (*P* < 0.01)

First, based on the coding DNA sequence region sequence of IL-17RA, sgRNAs were designed and validated for cleavage efficiency. Through SSA screening and Sanger sequencing, sgRNA2 and sgRNA6 were selected for generating *IL-17RA*-KO EPH4 (Fig. 1A–C). Subsequently, CCK-8 analysis demonstrated no difference in cell viability between *IL-17RA*-KO EPH4 and control EPH4 (Fig. 1D), and IL-17RA protein expression was not found in the knockout cells (Fig. 1E), confirming their suitability for subsequent experimental validation.

Second, results showed that compared to the LPS-only group, treatment with rIL-17A significantly up-regulated mRNA and protein expression levels of nuclear factor-κB activator 1 (ACT1), TNF receptor associated factor 6 (TRAF6), phospho-NF-kappa B inhibitor alpha (p-IKBα), phospho-Nuclear factor-κB (p-NF-κB), phospho-IκB kinase β (p-IKKβ), Phospho-p38 (p-p38), phospho-extracellular regulated protein kinases (p-ERK), interleukin-6 (IL-6), TNFα and Interleukin-1β (IL-1β), while zonula occludens-1 (ZO-1) and Occludin mRNA and protein expression were significantly down-regulated (*P* < 0.05; Fig. 1F–R and Fig. S1). This indicates that IL-17 pathway activation exacerbates inflammatory responses and disrupts TJ protein expression, resulting in TJ damage.

Notably, compared to the group treated with LPS and rIL-17A combined, the *IL-17RA*-KO EPH4 group exhibited significantly down-regulated mRNA and protein expression levels of ACT1, TRAF6, p-IKBα, p-NFκB, p-IKKβ, p-p38, p-pERK, IL-6, TNFα, and IL-1β, while ZO-1 and Occludin mRNA and protein expression were significantly up-regulated (*P* < 0.05; Fig. 1F–R and Fig. S1). This indicates that in the LPS-induced cell inflammation models, the IL-17RA-mediated IL-17 signaling pathway is a key factor in exacerbating inflammatory responses and TJ damage, and IL-17RA deletion effectively alleviates related damage. In conclusion, the IL-17RA-mediated IL-17 signaling pathway is a key factor in exacerbating the inflammatory response.

### *IL-17RA*^*−/−*^mice reduce the severity of mastitis

**Fig. 2:**
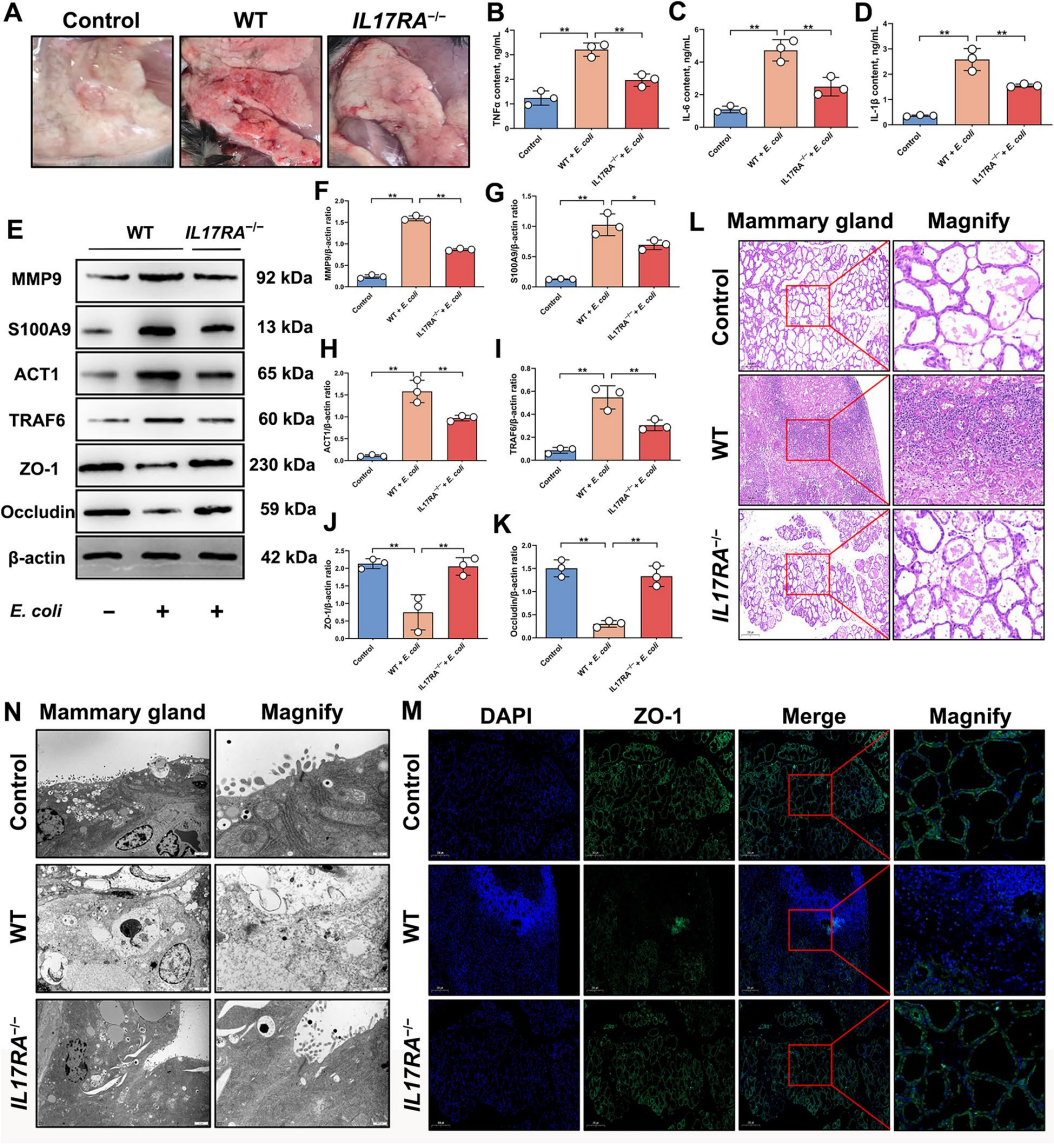
*IL-17RA*^−/−^ mice exhibit the ability to alleviate mastitis. **A** The mouse mammary gland appearance diagram for different treatment groups. **B** Analysis of TNFα protein content in mammary gland. **C** Analysis of IL-6 protein content in mammary gland. **D** Analysis of IL-1β protein content in mammary gland. **E** Detection of MMP9, S100A9, ACT1, TRAF6, ZO-1 and Occludin protein expression levels. **F** Analysis of MMP9 and β-actin protein expression levels. **G** Analysis of S100A9 and β-actin protein expression levels. **H** Analysis of ACT1 and β-actin protein expression levels. **I** Analysis of TRAF6 and β-actin protein expression levels. **J** Analysis of ZO-1 and β-actin protein expression levels. **K** Analysis of Occludin and β-actin protein expression levels. **L** HE analysis of mammary tissue from mice in different treatment groups. Bar = 200 µm. **M** IF analysis of ZO-1 expression in mammary tissue from mice in different treatment groups. Bar = 200 µm. **N** Transmission electron microscope analysis of mouse mammary tissue from different treatment groups. Bar = 2 µm or 500 nm. Values are expressed as mean ± SEM (*n* = 3 per group), * indicates significant difference (*P* < 0.05), ** indicates highly significant difference (*P* < 0.01)

To clarify the key role of the IL-17 signaling pathway in the pathogenesis of mastitis in vivo, this study established an in vivo mastitis model by infecting the mammary glands of *IL7RA*^−/−^ mice with *E. coli*. Results showed that, compared to the control group, *E. coli*-induced mastitis in wild-type mice resulted in mammary gland redness, swelling, and inflammation (Fig. 2A), and mRNA and protein expression levels of the proinflammatory factors IL-6, TNFα and IL-1β were significantly up-regulated (*P* < 0.01; Fig. 2B–D and Fig. S2A–C), indicating successful establishment of the mastitis model. However, compared to the *E. coli*-infected group, *IL-17RA*^−/−^ mice exhibited no significant redness or swelling in the mammary glands, and mRNA and protein expression levels of the pro-inflammatory factors IL-6, TNFα and IL-1β were significantly down-regulated (*P* < 0.01; Fig. 2B–D and Fig. S2A–C). This indicates that *IL-17RA*^−/−^ mice exhibit a marked resistance to mastitis induced by *E. coli* infection.

To further investigate the activation of the IL-17 signaling pathway during mastitis in mice, the expression levels of key proteins in this pathway were detected. Results showed that, compared to the control group, mRNA and protein expression levels of matrix metallopeptidase 9 (MMP9), ACT1, TRAF6, and s100 calcium binding protein A9 (S100A9) were significantly up-regulated in wild-type mice after *E. coli*-induced mastitis (*P* < 0.01; Fig. 2E–I and Fig. S2D–G). This indicates significant activation of the IL-17 signaling pathway during *E. coli*-induced mastitis. However, compared to the *E. coli*-infected group, mRNA and protein expression levels of MMP9, ACT1, TRAF6, and S100A9 were significantly down-regulated in the mammary glands of *IL-17RA*^−/−^ mice (*P* < 0.01; Fig. 2E–I and Fig. S2D–G). This indicates that IL-17RA deletion inhibited the activation of the IL-17 signaling pathway during mastitis in mice.

Notably, during mastitis, mRNA and protein expression levels of ZO-1 and Occludin were significantly up-regulated in the mammary glands of *IL-17RA*^−/−^ mice (*P* < 0.01; Fig. 2E, J–K and Fig. S2H–I). Further analysis using HE staining and IF revealed, compared to the *E. coli*-infected group, the mammary gland of *IL-17RA*^−/−^ mice exhibited a more intact BMB structure without massive accumulation of immune cells, and ZO-1 distribution within the mammary gland was uniform and normal (Fig. 2L–M). Furthermore, transmission electron microscope analysis confirmed the presence of intact TJ structures between mammary epithelial cells (Fig. 2N).

In conclusion, activation of the IL-17 signaling pathway during bacterial-induced mammary mastitis in mice represents a key pathway leading to exacerbated inflammatory responses and BMB structural damage within the mammary gland, while IL-17RA deletion effectively mitigates this pathological process, indicating IL-17RA as a potential therapeutic target.

### Multi-omics analysis revealed significantly activated IL-17 and TNF signaling pathways during mastitis

Based on in vivo and in vitro results, it has been demonstrated that inhibiting the IL-17 signaling pathway mediated by IL-17RA serves as a key therapeutic target for mastitis. However, the specific mechanisms by which the IL-17 signaling pathway mediates inflammatory amplification and barrier damage remain unclear. Therefore, we further investigated this using multi-omics integrated analysis after establishing a mouse mastitis model via *E. coli* infection.

**Fig. 3:**
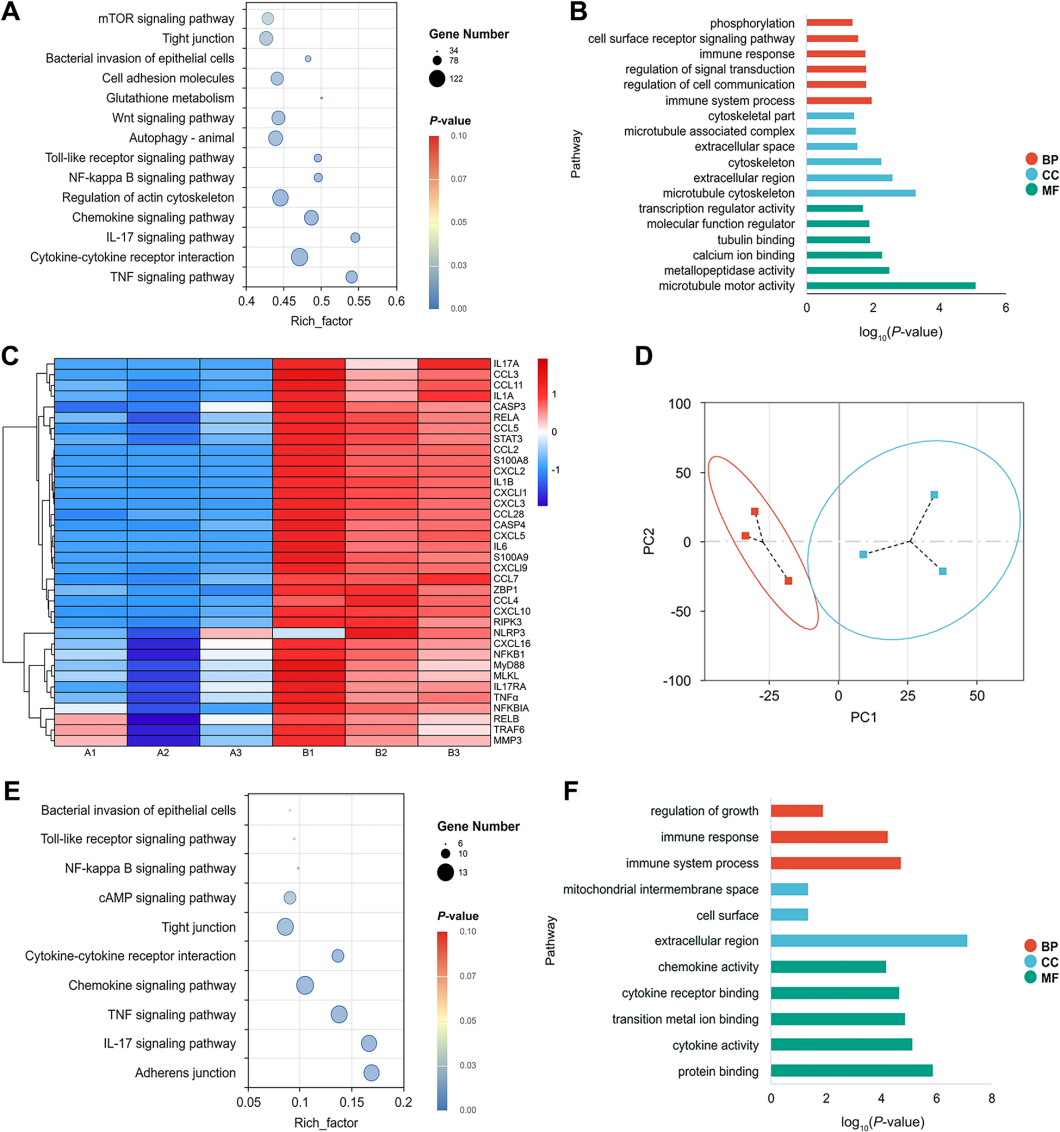
Multi-omics analysis reveals that IL-17 and TNF signaling pathways are key participants during mastitis. **A **Bubble maps obtained by KEGG analysis of RNA-seq data from groups A and B (A represents the control group and B represents the mastitis group), where the horizontal coordinate is the rich factor. **B** Bar graphs obtained by GO analysis of RNA-seq data from groups A and B. **C** Heat map analysis of RNA-seq data from groups A and B. **D** PCA analysis. **E** Bubble maps obtained by KEGG analysis of proteome sequencing data from groups A and B, where the horizontal coordinate is the rich factor. **F** Bar graphs obtained by GO analysis of proteome sequencing data from groups A and B

KEGG analysis of the RNA-seq revealed significant enrichment of pathways associated with inflammation and immune responses during the course of mouse mastitis, including mTOR signaling pathway, bacterial invasion of epithelial cells, Wnt signaling pathway, Toll-like receptor signaling pathway, NF-κB signaling pathway, IL-17 signaling pathway, TJ, and TNF signaling pathway (Fig. 3A). GO analysis further revealed significant enrichment in biological processes such as cell surface receptor signaling pathways, immune responses, immune system processes, calcium ion binding, and transcriptional regulation activation (Fig. 3B). Furthermore, heatmap analysis revealed that during mastitis, the following genes exhibited increased expression, including *IL-17A*, c–c motif chemokine ligand 3, c-c motif chemokine ligand 11, interleukin 1 alpha, cysteinyl aspartate specific proteinase 3, RELA proto-oncogene, NF-KB subunit, c–c motif chemokine ligand 5, signal transducer and activator of transcription 3, c–c motif chemokine ligand 2, s100 calcium binding protein A8, c-x-c motif chemokine ligand 2, *IL1β*, c-x-c motif chemokine ligand 11, c-x-c motif chemokine ligand 3, c–c motif chemokine ligand 28, cysteinyl aspartate specific proteinase 4, c-x-c motif chemokine ligand 5, *IL6*, *S100A9*, c-x-c motif chemokine ligand 19, c-c motif chemokine ligand 7, *ZBP1*, c-c motif chemokine ligand 4, c-x-c motif chemokine ligand 10 receptor interacting serine/threonine kinase 3, NLR family pyrin domain containing 3, c-x-c motif chemokine ligand 16, nuclear factor kappa B subunit 1, MYD88 innate immune signal transduction adaptor, mixed lineage kinase domain like pseudokinase, *IL-17RA*, *TNFα*, NFKB inhibitor alpha, RELB proto-oncogene, NF-KB subunit, *TRAF6* and matrix metallopeptidase 3 (Fig. 3C).

Proteome sequencing revealed different clustering patterns through principal component analysis. The first principal component partitioned the control group, while the second principal component partitioned the mastitis group, indicating data reproducibility (Fig. 3D). KEGG analysis results indicated significant enrichment in biological processes, including bacterial invasion of epithelial cells, Toll-like receptor signaling pathways, NF-κB signaling pathways, TJ, adhesion junctions, chemokine signaling pathways, cytokine-cytokine receptor interaction, IL-17 signaling pathways and TNF signaling pathways (Fig. 3E). GO analysis further revealed significant enrichment in biological processes, including immune responses, immune system processes, cell surface, cytokine receptor binding, cytokine activity and protein binding (Fig. 3F).

In conclusion, multi-omics sequencing results again confirm that the IL-17 signaling pathway is a key contributor during mastitis, exacerbating disease severity, while the TNF signaling pathway is also involved.

### IL-17 and TNF signaling pathways synergistically exacerbate inflammatory damage during mastitis

**Fig. 4:**
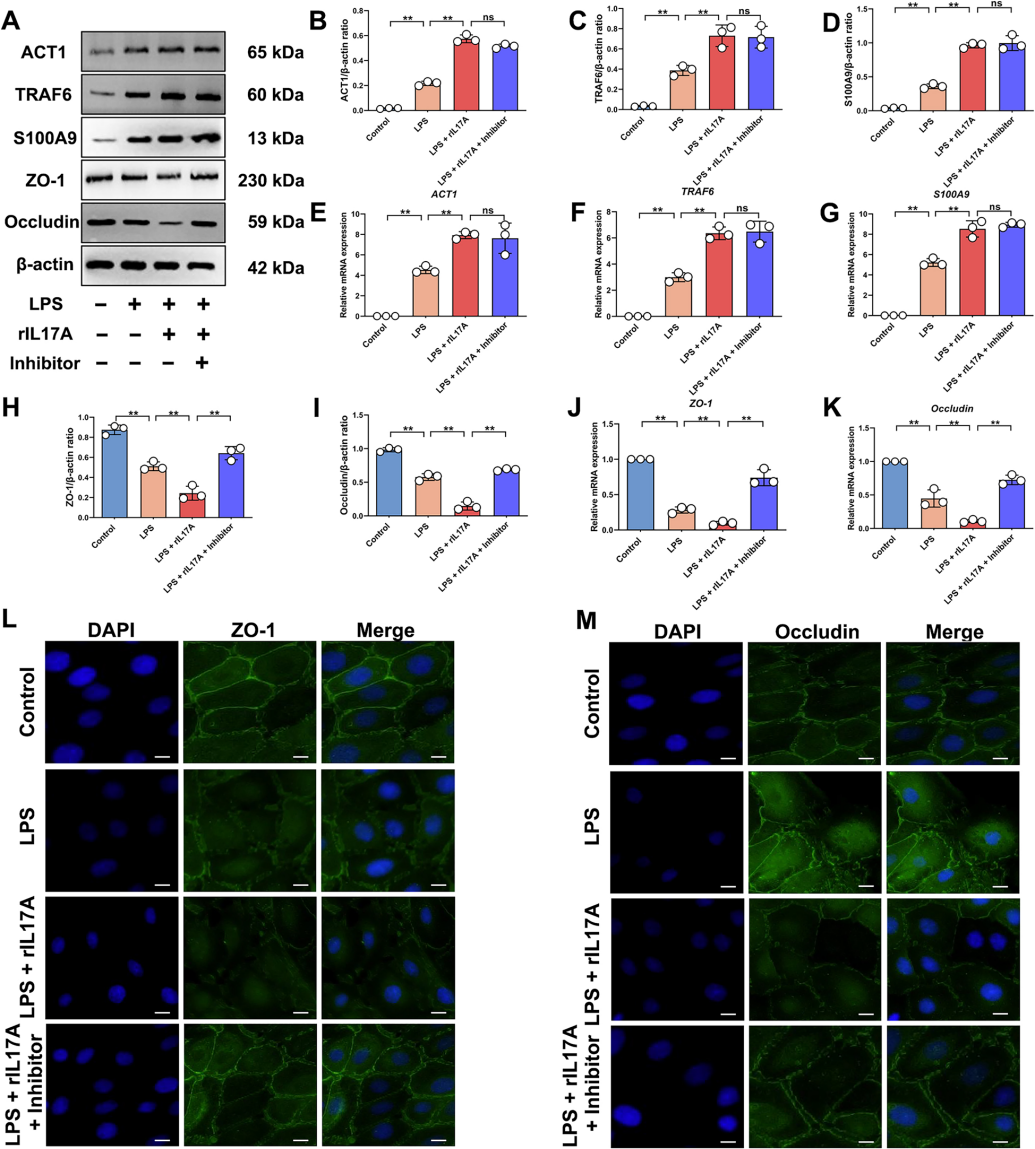
IL-17 and TNF signaling pathways synergistically exacerbate inflammatory responses and TJ damage. **A **Detection of ACT1, TRAF6, S100A9, ZO-1 and Occludin protein expression levels. **B** Analysis of ACT1 and β-actin protein expression levels. **C** Analysis of TRAF6 and β-actin protein expression levels. **D** Analysis of S100A9 and β-actin protein expression levels. **E** Analysis of *ACT1* mRNA expression levels. **F** Analysis of *TRAF6* mRNA expression levels. **G** Analysis of *S100A9* mRNA expression levels. **H** Analysis of ZO-1 and β-actin protein expression levels. **I** Analysis of Occludin and β-actin protein expression levels. **J** Analysis of *ZO-1* mRNA expression levels. **K** Analysis of *Occludin* mRNA expression levels. **L** IF analysis of ZO-1 expression on the cell membrane. Bar = 20 µm. **M** IF analysis of Occludin expression on the cell membrane. Bar = 20 µm. Values are expressed as mean ± SEM (*n* = 3 per group), * indicates significant difference (*P* < 0.05), ** indicates highly significant difference (*P* < 0.01)

To elucidate the relationship between these two signaling pathways during mastitis, validation was performed by treating EPH4 with LPS, rIL-17A and TNFα inhibitors. Results showed that, compared to the combined LPS and rIL-17A group, inhibiting TNFα expression resulted in no significant differences in ACT1, TRAF6 and S100A9 protein and mRNA expression levels (*P* > 0.05; Fig. 4A–G), but significantly upregulated ZO-1 and Occludin protein and mRNA expression (*P* < 0.01; Fig. 4A, H–K). This indicates that the TNF signaling pathway synergistically contributes to barrier structural damage during inflammation induced by IL-17 pathway activation. Subsequent IF analysis further showed that, compared to the combined LPS and rIL-17A group, inhibiting TNFα expression effectively maintained the distribution and structural integrity of ZO-1 and Occludin on the cell membrane (Fig. 4L and M). The above results showed that IL-17 and TNF signaling pathways synergistically promote mastitis progression. While inhibiting the TNF signaling pathway did not affect IL-17 pathway activation, it effectively mitigated TJ damage. Furthermore, this study also confirmed that IL-17RA deletion enhances mice's mastitis resistance. In conclusion, during bacterial mastitis, significant activation of the IL-17 signaling pathway promotes excessive activation of the TNF signaling pathway, thereby exacerbating the severity of mastitis and BMB damage.

### IL-17RA is a key target for baicalin's action

Based on a research strategy targeting IL-17RA protein as a potential therapeutic target for mastitis, this study employed network pharmacology screening and MD simulation methods to select and evaluate small-molecule compounds for target-specific binding to IL-17RA protein. Network pharmacology screening results indicated baicalin exhibits stable binding to IL-17RA protein (Table S6).

**Fig. 5:**
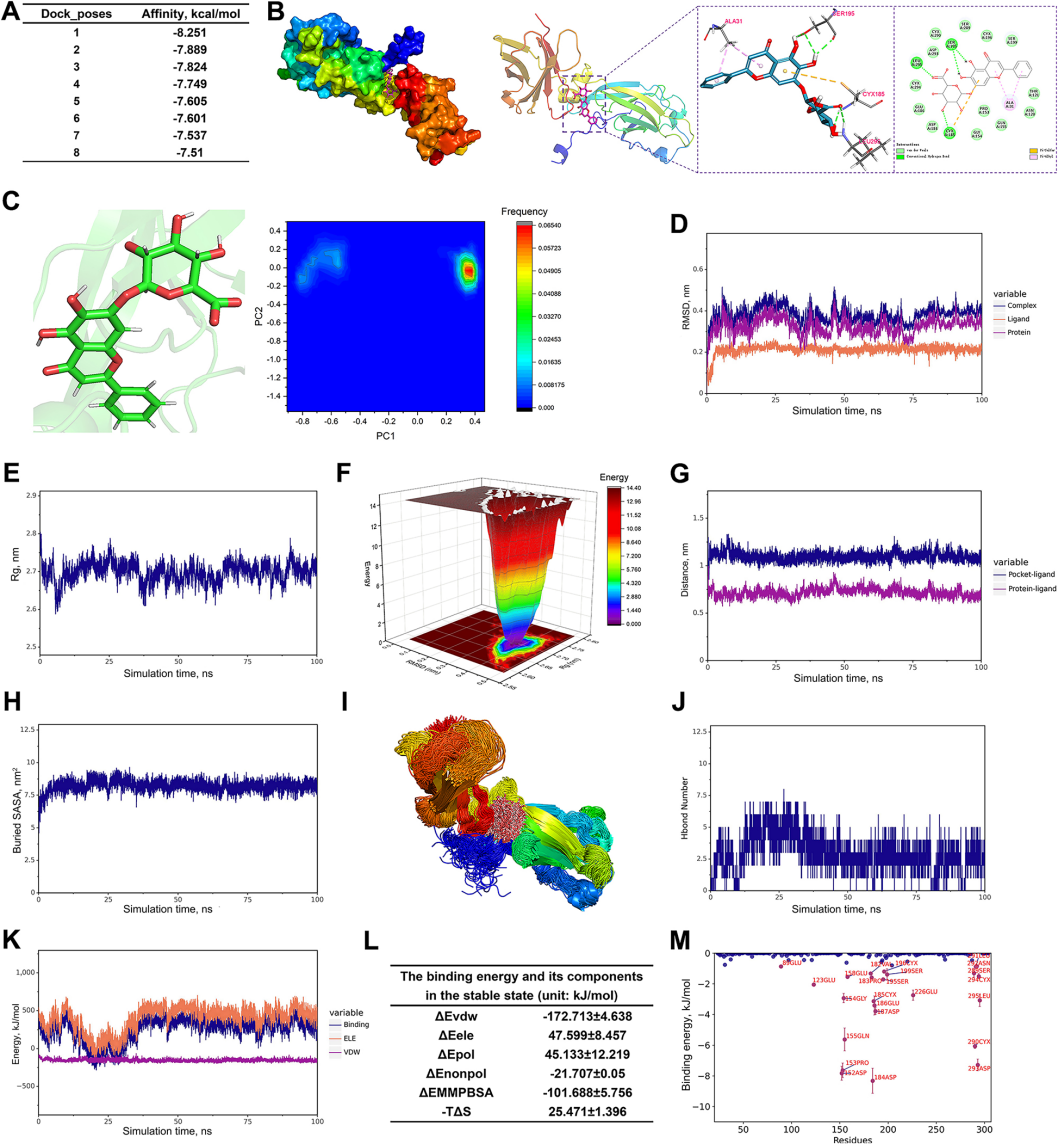
MD analysis indicates IL-17RA was the key target of baicalin. **A **Binding affinity analysis of baicalin and IL-17RA protein. **B** Interaction analysis between baicalin and IL-17RA protein. **C** Principal component analysis. **D** Root mean square deviation analysis of the complex, IL-17RAprotein and baicalin. **E** Radius of gyration analysis of the complex. **F** Free energy landscape analysis. **G** Dock site-ligand distance analysis between IL-17RA protein and baicalin. **H** Buried solvent accessible surface area analysis between IL-17RA protein and baicalin. **I** Conformation superposition analysis. **J** Hydrogen bond number analysis. **K** VDW and ELE binding energy analysis between IL-17RA protein and baicalin. **L** Binding energy and its components at equilibrium (unit: kJ/mol). **M** Amino acid contribution to binding energy analysis

To comprehensively evaluate the binding affinity and stability between baicalin and IL-17RA protein, an affinity analysis was conducted, and the results revealed a strong affinity between them (Fig. 5A). Specifically, amino acid residues SER-195, LEU-295 and CYS-185 in the IL-17RA protein formed hydrogen bonds with baicalin, while CYS-185 and ALA-31 formed Pi-sulfur and Pi-alkyl hydrophobic interactions with baicalin, respectively. Additionally, residues such as SER-289, GLU-186 and ASN-120 form van der Waals (VDW) interactions with baicalin (Fig. 5B). Based on this binding pattern, we further conducted MD simulations to validate the binding stability between baicalin and the IL-17RA protein, and to analyze its hydrogen bonds and other interactions.

First, principal component analysis and surface electrostatic potential analysis revealed that the scutellarin conformation remained stable throughout the simulation (Fig. 5C), exhibiting negative charge on the surface of the IL17RA protein binding site (Fig. S3A). Root mean square deviation and radius of gyration reveal that the protein–ligand complex structure progressively stabilises (Fig. 5D–E) and exhibits a low-energy state (Fig. 5F), suggesting structural stability of the baicalin-IL17RA protein complex. Second, Root-mean-square fluctuation and center-of-mass evolution analysis revealed low flexibility in amino acid residues surrounding baicalin (Fig. S3B), while the distance between baicalin and the IL-17RA protein center, as well as its spacing from the binding site, gradually stabilized. This further indicates the progressive stabilization of their binding state (Fig. 5G). Buried solvent accessible surface area analysis also revealed gradual stabilization of this value, suggesting that the contact area between baicalin and IL-17RA proteins stabilized, indicating a stable binding state (Fig. 5H). Notably, conformation superposition analysis of the simulation trajectories revealed that baicalin consistently bound to the same region of the IL-17RA protein throughout the simulation, exhibiting high superposition accuracy (Fig. 5I). Finally, Hydrogen bond frequency analysis revealed multiple high-frequency hydrogen bond pairs between baicalin and IL-17RA protein, such as the 120 ASN:Ligand pair with an occurrence frequency of 57.6%, indicating strong binding stability (Fig. 5J and Fig. S3C). Electrostatic (ELE) and VDW force analysis further revealed that VDW and ELE interactions in the complex remained stable throughout the simulation, indicating sustained stability in the small molecule-protein binding (Fig. 5K). In the steady state, the binding free energy ΔEMMPBSA between baicalin and IL-17RA protein was −101.688 ± 5.756 kJ/mol, indicating high binding affinity between the two (Fig. 5L). Notably, the free energy decomposition analysis revealed that key residues in the protein, such as ASP-184 and ASP-152, significantly contributed to the binding energy (Fig. 5M).

In conclusion, baicalin and IL-17RA protein form a stable complex where VDW interactions play a primary role in binding energy, hydrophobic interactions contribute secondarily and ELE interactions provide supplementary effects.

### Baicalin alleviated the severity of mastitis in mice by inhibiting IL-17 signaling pathway activation

**Fig. 6:**
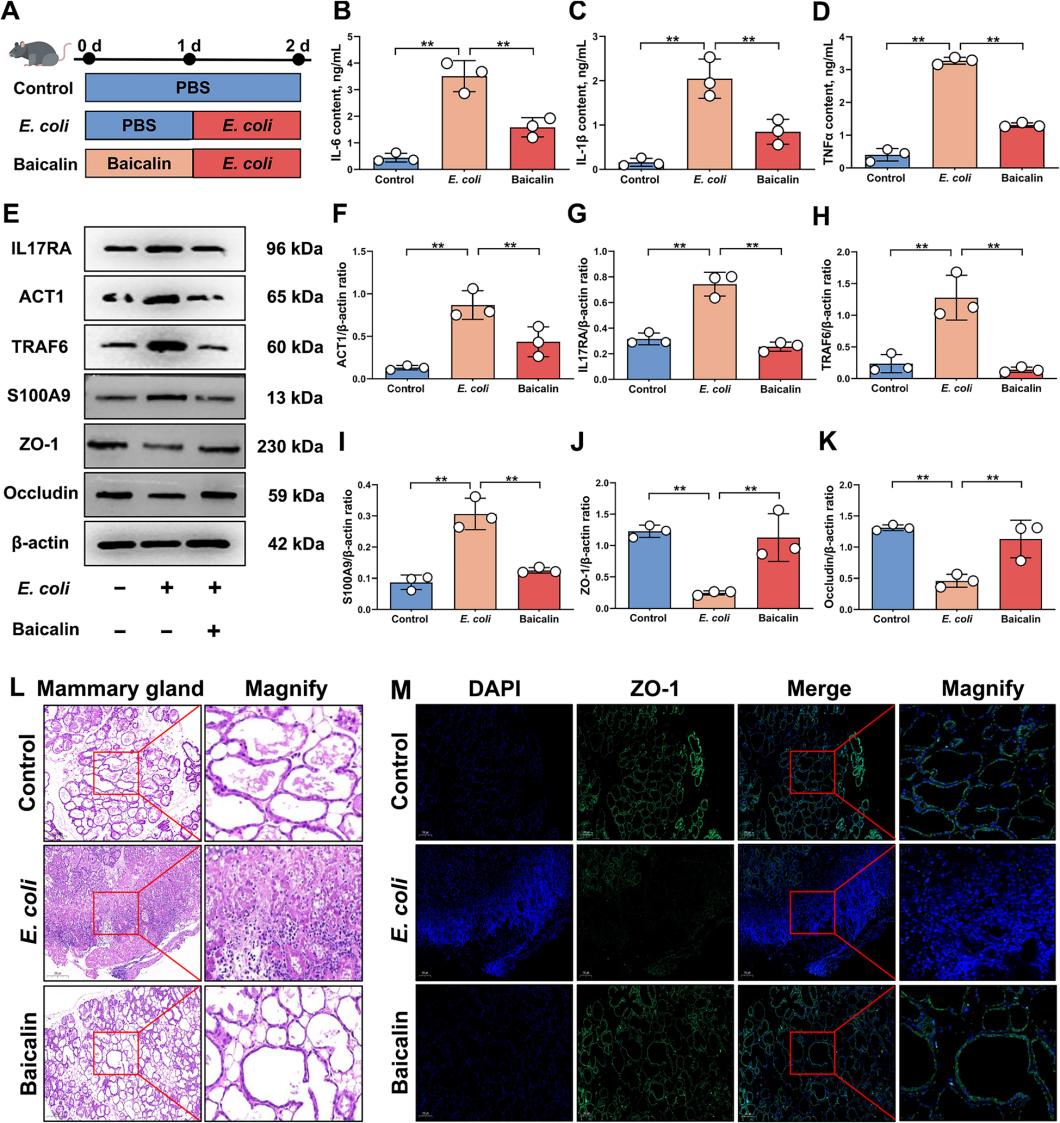
Baicalin inhibits IL-17 signaling pathway activation to reduce the severity of mastitis in mice. **A** Schematic diagram of mastitis models constructed in mice. **B** Analysis of IL-6 protein content in mammary gland. **C** Analysis of IL-1β protein content in mammary gland. **D** Analysis of TNFα protein content in mammary gland. **E** Detection of ACT1, IL-17RA, TRAF6, S100A9, ZO-1 and Occludin protein expression levels. **F** Analysis of ACT1 and β-actin protein expression levels. **G** Analysis of IL-17RA and β-actin protein expression levels. **H** Analysis of TRAF6 and β-actin protein expression levels. **I** Analysis of S100A9 and β-actin protein expression levels. **J** Analysis of ZO-1 and β-actin protein expression levels. **K** Analysis of Occludin and β-actin protein expression levels. **L** HE analysis of mammary tissue from mice in different treatment groups. Bar = 200 µm. **M** IF analysis of ZO-1 expression in mammary tissue from mice in different treatment groups. Bar = 100 µm. Values are expressed as mean ± SEM (*n* = 3 per group), * indicates significant difference (*P* < 0.05), ** indicates highly significant difference (*P* < 0.01)

Although baicalin can treat various inflammation-related diseases, its effects on mastitis and detailed molecular mechanisms remain unclear. Therefore, based on the findings of this study, it is speculated that baicalin may alleviate mastitis by regulating the IL-17RA-mediated IL-17 signaling pathway. Accordingly, a mouse mastitis model induced by *E. coli* infection was established and treated with baicalin for further investigation (Fig. 6A). Results showed that compared to the mastitis group, baicalin treatment significantly reduced mRNA and protein expression levels of IL-6, TNFα, IL-1β, IL-17RA, ACT1, TRAF6 and S100A9 (*P* < 0.01; Fig. 6B–I and Fig. S4A–G), while significantly up-regulated ZO-1 and Occludin expression (*P* < 0.01; Fig. 6E, J–K and Fig. S4H–I). This indicates that baicalin effectively suppresses IL-17 signaling pathway activation during mastitis and stabilizes TJ protein expression. Furthermore, HE and IF analyses revealed that compared to the mastitis group, the baicalin-treated group exhibited more intact mammary gland structures, reduced immune cell infiltration and ZO-1 protein distributed uniformly in the mammary glands (Fig. 6L–M). This indicates that baicalin alleviates BMB damage during mastitis. In conclusion, baicalin modulates IL-17RA-mediated IL-17 signaling pathway activation to reduce the severity of *E. coli*-induced mastitis.

### Baicalin alleviated the severity of mastitis in dairy cows by inhibiting IL-17 signaling pathway activation

**Fig. 7:**
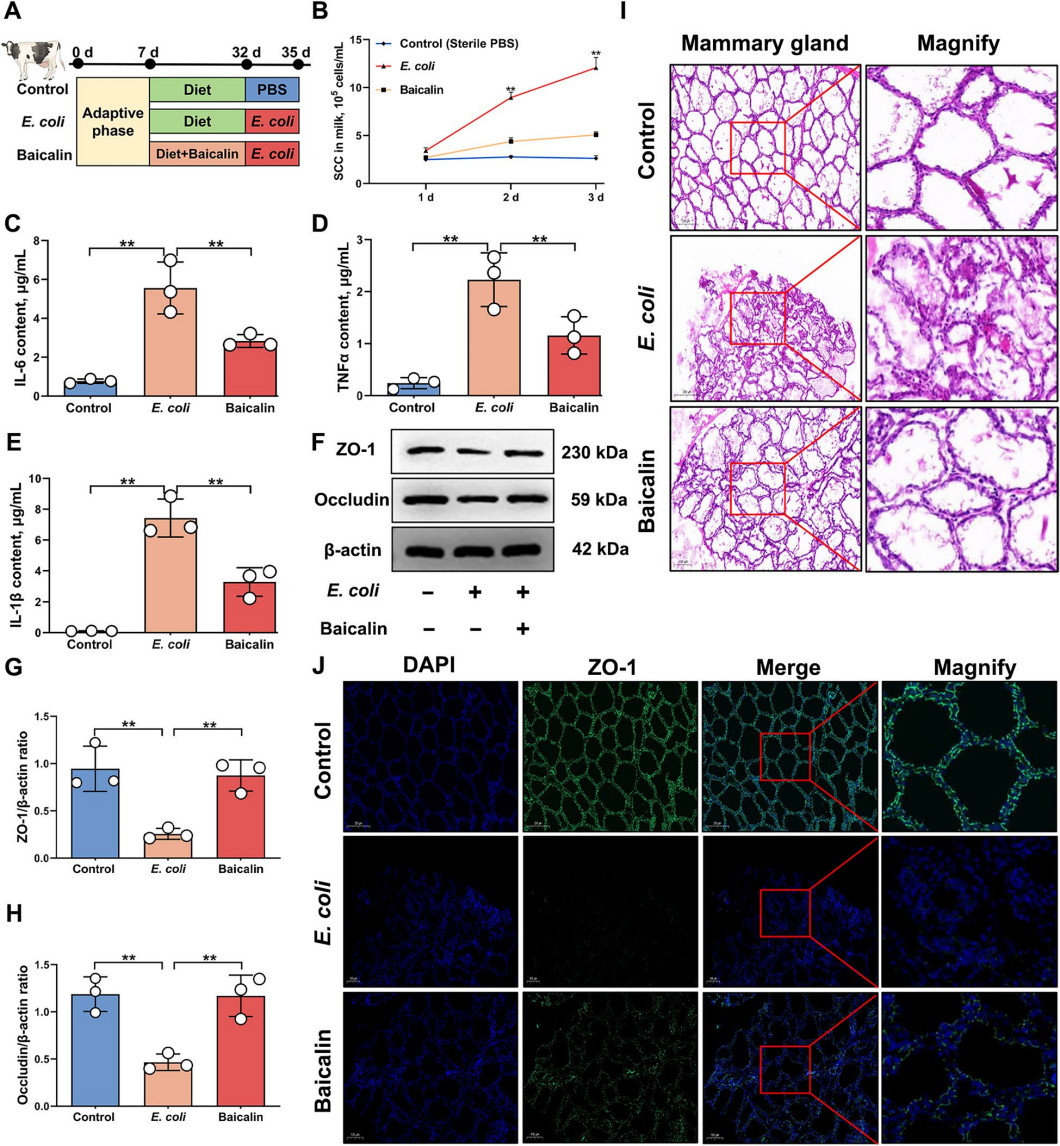
Baicalin effectively alleviates mastitis in dairy cows. **A** Schematic diagram of mastitis models constructed in dairy cows. **B** SCC indicator detection. **C** Analysis of IL-6 protein content in mammary gland. **D** Analysis of TNFα protein content in mammary gland. **E** Analysis of IL-1β protein content in mammary gland. **F** Detection of ZO-1 and Occludin protein expression levels. **G** Analysis of ZO-1 and β-actin protein expression levels. **H** Analysis of Occludin and β-actin protein expression levels. **I** HE analysis of mammary tissue from mice in different treatment groups. Bar = 200 µm. **J** IF analysis of ZO-1 expression in mammary tissue from mice in different treatment groups. Bar = 200 µm. Values are expressed as mean ± SEM (*n* = 3 per group), * indicates significant difference (*P* < 0.05), ** indicates highly significant difference (*P* < 0.01)

Baicalin has been validated for treating mastitis in mice, but its efficacy in dairy cows remains unclear. Therefore, a mastitis model in dairy cows was established using *E. coli* infection to evaluate baicalin's treatment effects (Fig. 7A). Results indicate that compared to the mastitis group, baicalin treatment reduced SCC and the expression levels of IL-6, TNFα and IL-1β during mastitis in dairy cows, while significantly up-regulated ZO-1 and Occludin expression (*P* < 0.01; Fig. 7B–H and Fig. S5). This suggests baicalin alleviates the severity of *E. coli*-induced mastitis in dairy cows. Subsequent, HE and IF analyses further showed that baicalin treatment maintained mammary gland structural integrity, reduced immune cell infiltration, and stabilized ZO-1 expression and normal distribution within the mammary gland (Fig. 7I–J). This indicates that baicalin alleviates BMB damage during bacterial mastitis in dairy cows.

**Fig. 8:**
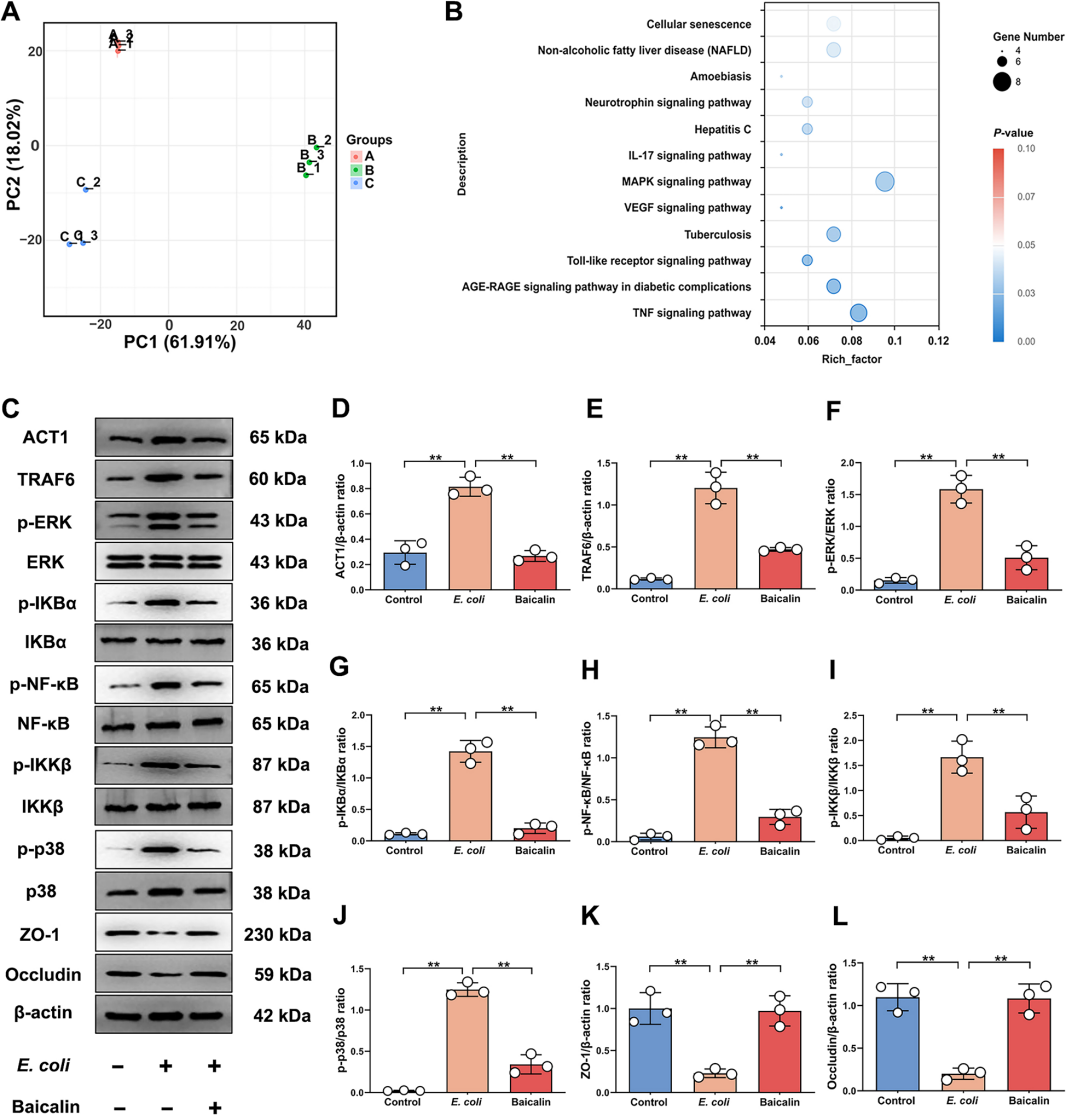
Baicalin inhibits IL-17 signaling pathway activation to reduce the severity of mastitis in dairy cows. **A** PCA Analysis (A represents the control group, B represents the mastitis group, and C represents the baicalin-treated group). **B** Bubble maps obtained by KEGG analysis of proteome sequencing data from groups B and C, where the horizontal coordinate is the rich factor. **C** Detection of ACT1, TRAF6, p-ERK, ERK, p-IKBα, IKBα, p-NF-κB, NF-κB, p-IKKβ, p-IKKβ, p-p38, p38, ZO-1 and Occludin protein expression levels. **D** Analysis of ACT1 and β-actin protein expression levels. **E** Analysis of TRAF6 and β-actin protein expression levels. **F** Analysis of p-ERK and ERK protein expression levels. **G** Analysis of p-IKBα and IKBα protein expression levels. **H** Analysis of p-NF-κB and NF-κB protein expression levels. **I** Analysis of p-IKKβ and IKKβ protein expression levels. **J** Analysis of p-p38 and p38 protein expression levels. **K** Analysis of ZO-1 and β-actin protein expression levels. **L** Analysis of Occludin and β-actin protein expression levels. Values are expressed as mean ± SEM (n = 3 per group), * indicates significant difference (*P* < 0.05), ** indicates highly significant difference (*P* < 0.01)

To further investigate whether the therapeutic effect of baicalin in dairy cows mastitis is related to the IL-17 signaling pathway, proteome sequencing of mammary gland tissue from dairy cows was performed for validation. The results revealed different clustering patterns through principal component analysis, the first principal component partitioned the control group, the second partitioned the inflammation group, and the third partitioned the baicalin-treated group, indicating data reproducibility (Fig. 8A). Subsequent KEGG analysis showed significant enrichment of the IL-17 signaling pathway and TNF signaling pathway, suggesting their involvement in baicalin's therapeutic process (Fig. 8B). Further analysis of key protein expression in the IL-17 signaling pathway revealed that baicalin treatment significantly down-regulated mRNA and protein expression of ACT1, TRAF6, p-IKBα, p-NFκB, p-p38, p-ERK and p-pIKKβ, while up-regulated ZO-1 and Occludin expression (*P* < 0.01; Fig. 8C–L and Fig. S6). This indicates that baicalin inhibits IL-17 signaling pathway activation during bovine mastitis.

In conclusion, baicalin effectively alleviates *E. coli*-induced mastitis in dairy cows by modulating the IL-17 signaling pathway to suppress inflammatory responses and maintain mammary barrier integrity.

## Discussion

Bacterial mastitis is one of the most common and highly destructive major diseases in animal husbandry, characterized by inflammatory imbalance within the mammary gland and disruption of the BMB. This disease not only severely hampers the sustainable development of animal husbandry, but also poses a threat to public health and safety [1, 2, 30]. Currently, effective treatment options for this disease remain limited, and antibiotics remain the primary therapeutic strategy [31]. Therefore, clarifying the key mechanisms underlying its pathogenesis and developing targeted therapeutic strategies based on this are essential to effectively mitigate the adverse impacts of this disease. Although the IL-17 signaling pathway has been identified as a key driver of inflammatory pathological processes, its specific mechanisms regulating the inflammatory cascade and mammary gland dysfunction in mastitis remain unclear. This study, comprehensively utilizing dairy cows and knockout mouse models, demonstrates that IL-17RA is a key factor exacerbating mastitis severity, and that genetic knockout or pharmacological inhibition of IL-17RA effectively suppressed the inflammatory response within the mammary gland and maintained BMB integrity, thereby alleviating disease progression. Furthermore, this study confirms that the natural flavonoid compound baicalin exerts therapeutic effects by regulating IL-17RA, as a potential antibiotic alternative. This finding also provides evidence that plant extracts represent a promising strategy for antibiotic replacement therapy.

**Fig. 9:**
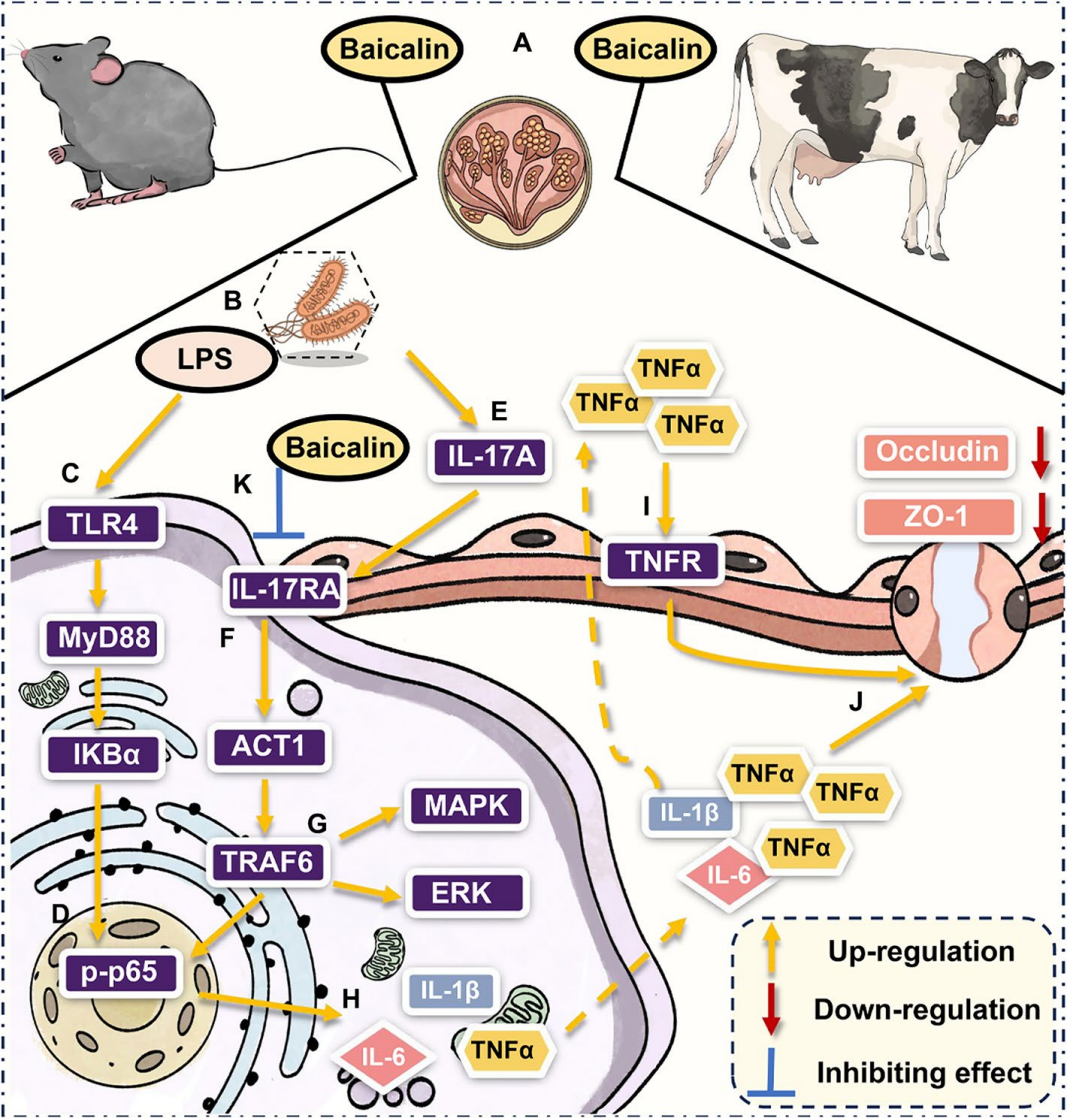
Schematic overview of baicalin's mechanism in alleviating mastitis by regulating the IL-17RA-mediated IL-17 signaling pathway. **A** Oral administration of baicalin to mice and dairy cows. **B** In vivo release of LPS by *E. coli*. **C** LPS activated the TLR4/MyD88/NF-κB pathway in epithelial cells. **D** NF-κB was activated within the cell nucleus. **E** *E. coli* infection increases IL-17A content in mammary glands. **F** IL-17A binds to IL-17RA on the cell membrane surface, thereby activating the IL-17 signaling pathway. **G** Activation of the MAPK signaling pathway and ERK signaling pathway. **H** Production of IL-6, TNFα and IL-1β. **I** TNFα activated TNF signaling pathway. **J** TJ structure damage. **K** Baicalin inhibits IL-17RA signal transduction, thereby preventing activation of the IL-17 signaling pathway

In mastitis, few studies have investigated the IL-17 signaling pathway, but high levels of IL-17A have been detected in mastitis cases, suggesting that this pathway plays a significant role in the disease [17, 32]. In this study, IL-17RA deletion caused mice to exhibit significant resistance to *E. coli*-induced mastitis, highlighting the core position of the IL-17 signaling pathway, as shown by significantly reduced clinical symptoms, reduced histopathological damage, and down-regulated expression of key proinflammatory cytokines (IL-6, TNFα and IL-1β), indicating that the massive release of proinflammatory factors was dependent on IL-17RA signaling transduction. In a mouse colitis model, suppression of IL-17RA expression effectively maintained epithelial barrier integrity and prevented disease progression [33, 34]. Notably, in an *IL-17RA*^−/−^ mouse mastitis model, ZO-1 and Occludin were stably expressed in mammary tissue, maintaining BMB structural integrity, providing direct genetic evidence for IL-17 signaling pathway activation as the primary mediator of BMB damage. Furthermore, upon binding to the IL-17RA receptor on target cell surfaces, IL-17A recruits ACT1 and TRAF6, subsequently activating downstream MAPK and NF-κB signaling pathways, resulting in massive release of proinflammatory factors, which exacerbates inflammatory responses and was confirmed in this study [35, 36]. Based on this, further in vitro experiments using mammary epithelial cells confirmed this causal relationship, IL-17A synergistically with LPS excessively activated the MAPK signaling pathway, ERK signaling pathway and NF-κB signaling pathway, increasing the production of inflammatory cytokines and disrupting TJ integrity, and these phenomena were effectively reversed after IL-17RA deletion. This demonstrated that during *E. coli*-induced mastitis, released LPS not only activates the classical TLR4/MyD88/NF-κB pathway to produce proinflammatory factors [29], but also that IL-17A activates IL-17RA signaling to promote the production of inflammatory cytokine storm. In conclusion, this confirms that IL-17RA activation is the core factor for exacerbating the severity of mastitis and BMB damage (Fig. 9).

TNFα is a key factor in exacerbating inflammatory responses and disrupting the immune microenvironment, contributing to the occurrence of a cytokine storm within the mammary gland [37, 38]. In this study, multi-omics sequencing analysis revealed that the IL-17 signaling pathway and TNF signaling pathway were critical participants in the development of mastitis. Furthermore, relevant studies have confirmed that the IL-17 signaling pathway and TNF signaling pathway are key driving mechanisms in various inflammatory diseases, this suggests both pathways play a central role in mastitis disease [39, 40]. Based on this, this study further investigated and confirmed the existence of a key synergistic relationship between these two pathways, demonstrating that IL-17RA deletion effectively down-regulated TNFα expression, but inhibition of the TNF signaling pathway could not directly block IL-17 signaling pathway activation (e.g., ACT1 and TRAF6 expression levels). Moreover, inhibiting the TNF signaling pathway alleviated BMB damage during mastitis. This revealed a molecular regulatory network in mastitis progression, where IL-17A acts as an upstream driver, activating IL-17RA to promote IL-17 signaling pathway activation and causing excessive TNFα expression and over-activation the TNF signaling pathway to exert synergistic effects, thereby exacerbating inflammatory responses and causing related damage. Importantly, this also explains why IL-17RA deletion could effectively suppress the inflammatory cytokine storm and alleviate BMB structural damage within the mammary gland. In conclusion, the IL-17 signaling pathway and TNF signaling pathway synergistically exacerbate inflammatory responses and cause BMB damage in mastitis, resulting in disease progression (Fig. 9).

Along with intensive research on mastitis, plant extracts have emerged as a promising therapeutic strategy due to their safety and efficacy [2, 5]. Relevant studies reported that baicalin was an effective flavonoid compound for treating inflammatory diseases, and IL-17RA was an effective therapeutic target for immune-mediated diseases [41, 42]. Based on this, given the pivotal role of IL-17RA in mastitis, this study employed MD simulations to reveal a high affinity between baicalin and IL-17RA, demonstrating in vivo and in vitro mastitis models, baicalin effectively modulates IL-17 signaling pathway activation, reduces pro-inflammatory factor release, and preserves barrier integrity. Importantly, in both mouse and dairy cow mastitis models, baicalin treatment significantly reduced inflammatory responses and BMB damage within mammary gland tissues. Furthermore, proteomics analysis confirmed that the IL-17 signaling pathway and TNF signaling pathway are significantly involved when baicalin exerts its mastitis-alleviating effects. In conclusion, the key mechanism by which baicalin demonstrates therapeutic potential for alleviating mastitis is achieved through regulating IL-17RA signaling transduction and subsequently blocking the inflammatory cascade reaction (Fig. 9).

Although this study provides a relatively comprehensive understanding of the mechanism by which baicalin alleviates mastitis, certain limitations remain. The study employed an *E. coli*-induced acute mastitis model, while *Staphylococcus aureus* is also a common pathogen causing mastitis. Therefore, whether baicalin exhibits equivalent protective effects against mastitis induced by this bacterium requires further validation.

## Conclusions

This study reveals that in mastitis, baicalin inhibits IL-17 signaling pathway activation by targeting IL-17RA and reduces excessive activation of the TNF signaling pathway, preventing exacerbation of inflammatory responses, thereby maintaining mammary gland immune homeostasis and protecting BMB integrity. Furthermore, both pharmacological inhibition via baicalin and IL-17RA deletion effectively alleviated mastitis severity, confirming an effective therapeutic target. This study not only provides novel insights into mastitis pathogenesis but also demonstrates baicalin as a promising targeted therapeutic agent and antibiotic alternative, holding significant implications for sustainable livestock production, animal welfare, and public health.

## Supplementary Information

Additional file 1. Original Western blot images.

Additional file 2. Fig. S1. Analysis of *ACT1*, *TRAF6*, *ERK*, *IKBα*, *NF-κB*, *IKKβ*, *p38*, *IL-6*, *IL-1β*, *TNFα*, *ZO-1* and *Occludin* mRNA expression levels. Fig. S2. Analysis of *IL-6*, *IL-1β*, *TNFα*, *MMP9*, *ACT1*, *TRAF6*, *S100A9*, *ZO-1* and *Occludin* mRNA expression levels. Fig.e S3. MD analysis of baicalin binding to IL-17RA. Fig. S4. Analysis of *IL-6*, *IL-1β*, *TNFα*, *IL-17RA*, *ACT1*, *TRAF6*, *S100A9*, *ZO-1* and *Occludin* mRNA expression levels. Fig. S5. *ZO-1* and *Occludin* mRNA expression levels. Fig. S6. Analysis of *ACT1*, *TRAF6*, *ERK*, *IKBα*, *NF-κB*, *IKKβ*, *p38*, *ZO-1* and *Occludin* mRNA expression levels. Table S1. Primers for RT-qPCR for mouse species. Table S2. Primers for RT-qPCR for bovine species. Table S3. Antibody information. Table S4. Mouse IL-17RA CDS sequence information. Table S5. SgRNA sequence information. Table S6. TCMBANK selection results.

## Data Availability

All raw data generated during the current study are available from the corresponding authors upon request.

## Abbreviations

ACT1: Nuclear factor-κB activator 1
BMB: Blood-milk barrier
*E. coli*: *Escherichia coli*
ELE: Electrostatic
ERK: Extracellular regulated protein kinases
HE: Hematoxylin-eosin
IF: Immunofluorescence
IL-17RA: Interleukin 17 receptor A
IL-1β: Interleukin-1β
IL-17: Interleukin 17
IL-6: Interleukin-6
KO: Knockout
MAPK: Mitogen-activated protein kinase
NF-κB: Nuclear factor kappa-B
p-ERK: Phospho-extracellular regulated protein kinases
p-IKBα: Phospho-NF-kappa B inhibitor alpha
p-IKKβ: Phospho-IκB kinase β
p-NFκB: Phospho-nuclear factor-κB
p-p38: Phospho-p38
rIL-17A: Recombinant IL-17A
SCC: Somatic cell count
S100A9: S100 calcium binding protein A9
TJ: Tight junctions
TNFα: Tumor necrosis factor-α
TRAF6: TNF receptor associated factor 6
VDW: Van der Waals
ZO-1: Zonula occludens-1
