# Supplementary material for: Baicalin alleviates mastitis in dairy cows by targeting IL-17RA to inhibit IL-17 signaling pathway activation
Source: J Anim Sci Biotechnol. 2026 Apr 16;17:69. doi: 10.1186/s40104-026-01401-2 (PMC13085363; doi:10.1186/s40104-026-01401-2)

Figure 1. ACT1

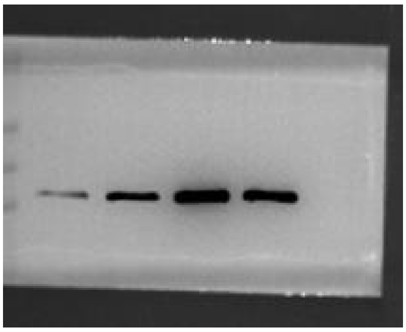

Figure 1. TRAF6

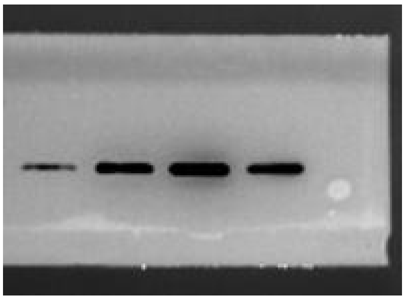

Figure 1. p-ERK

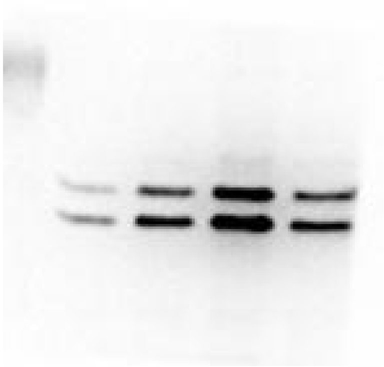

Figure 1. ERK

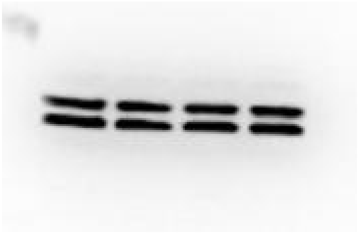

Figure 1. p-IKBα

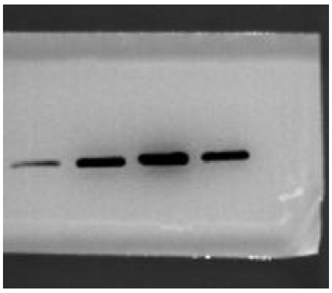

Figure 1. IKBα

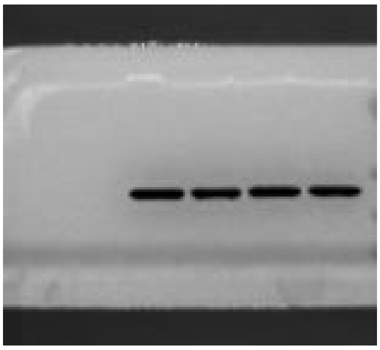

Figure 1. p-NF-κB

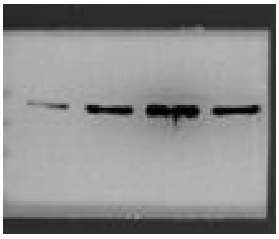

Figure 1. NF-κB

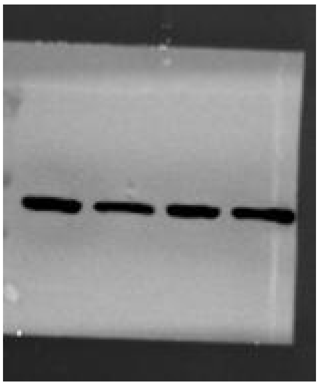

Figure 1. p-IKKβ

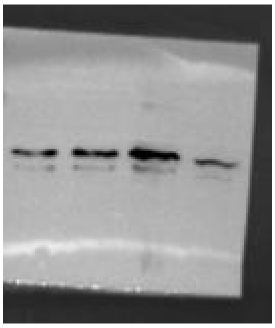

Figure 1. IKKβ

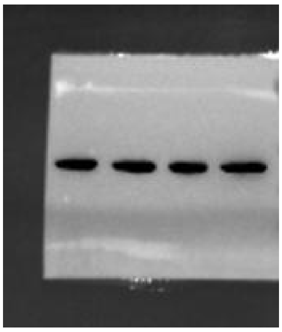

Figure 1. p-p38

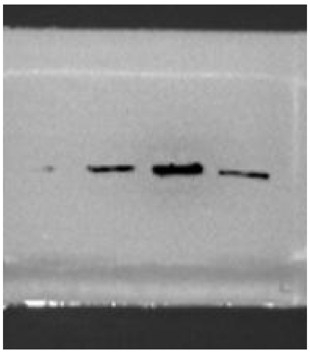

Figure 1. p38

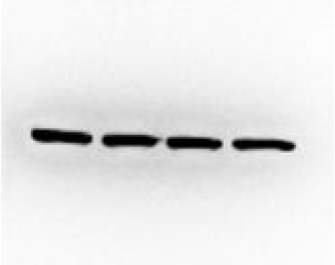

Figure 1. IL-6

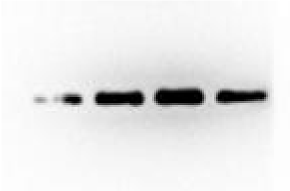

Figure 1. IL-1β

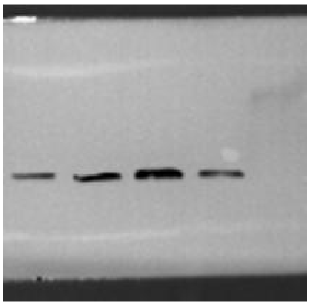

Figure 1. TNFα

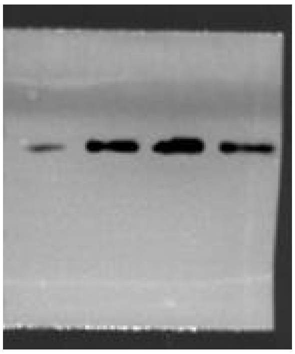

Figure 1. Occludin

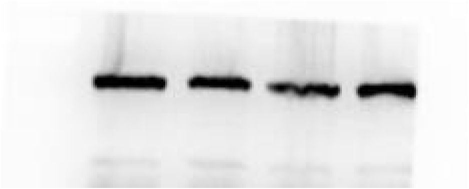

Figure 1. ZO-1

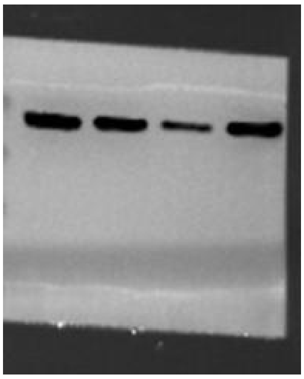

Figure 1. β-actin

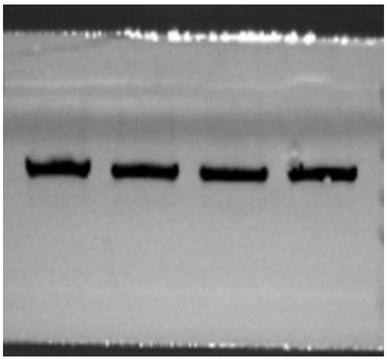

Figure 1. IL-17RA

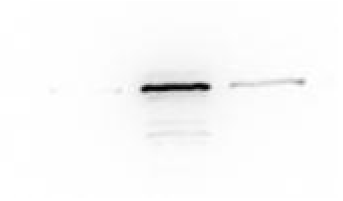

Figure 2. MMP9

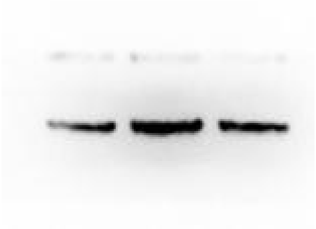

Figure 2.S100A9

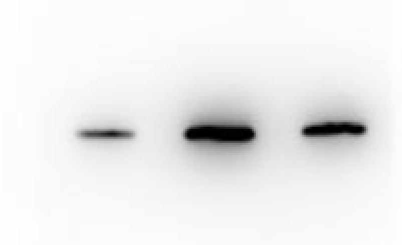

Figure 2. ACT1

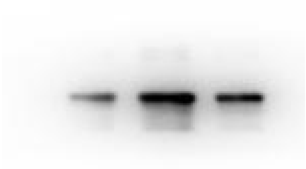

Figure 2. TRAF6

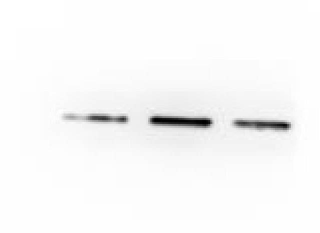

Figure 2. Occludin

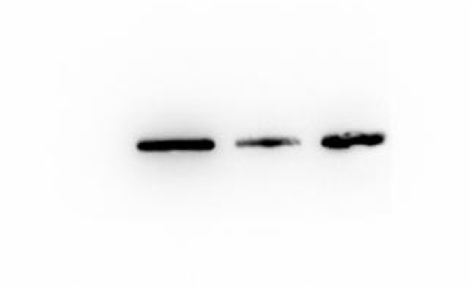

Figure 2. ZO-1

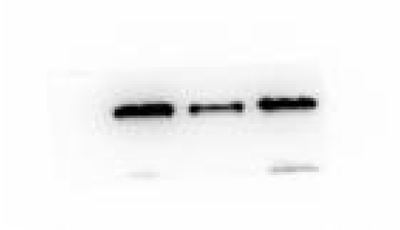

Figure 2. β-actin

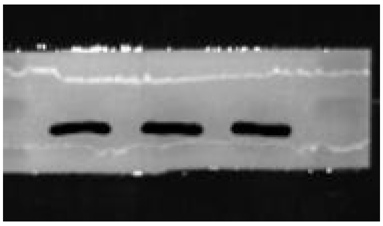

Figure 4. ACT1

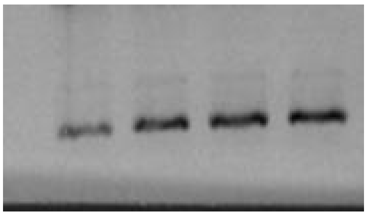

Figure 4. TRAF6

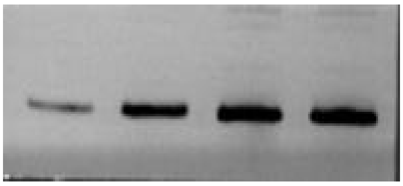

Figure 4. S100A9

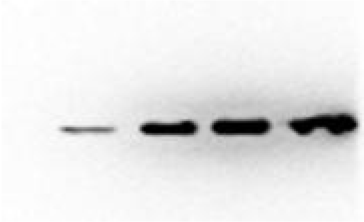

Figure 4. ZO-1

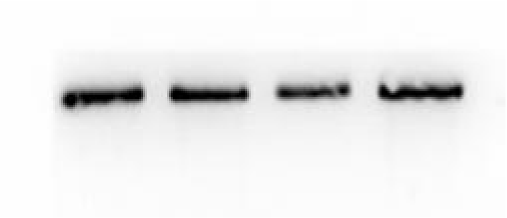

Figure 4. Occludin

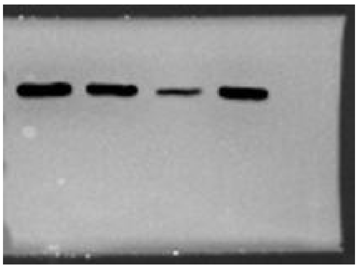

Figure 4. β-actin

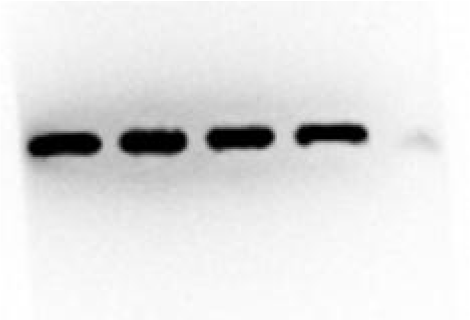

Figure 6. IL17RA

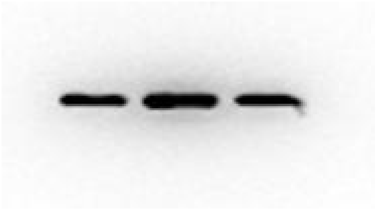

Figure 6. ACT1

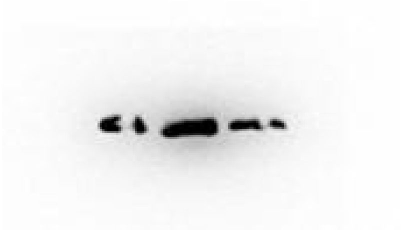

Figure 6. S100A9

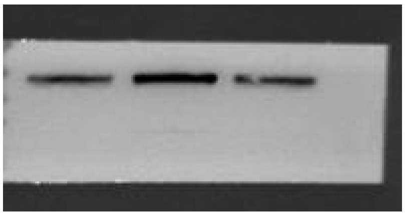

Figure 6. Occludin

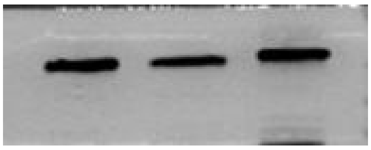

Figure 6. β-actin

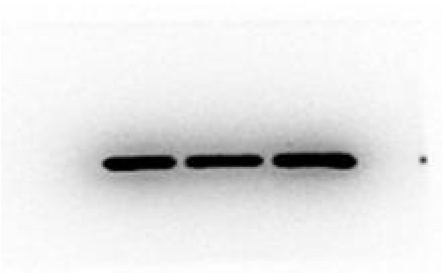

Figure 6. ZO-1

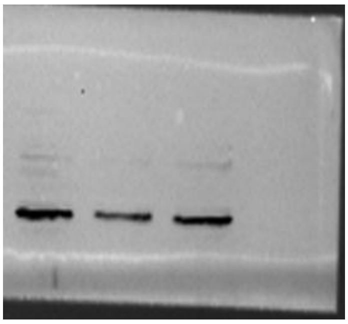

Figure 6. TRAF6

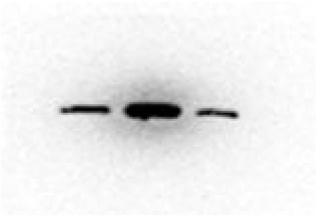

Figure 7. ZO-1

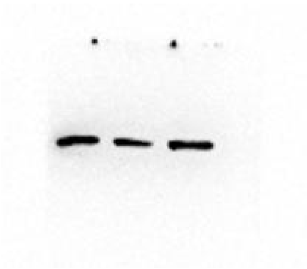

Figure 7. Occludin

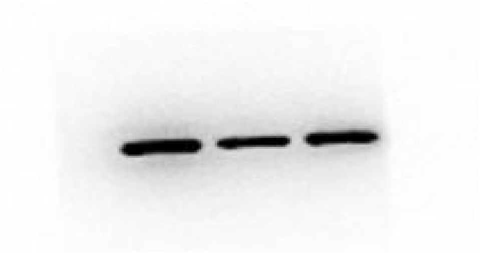

Figure 7. β-actin

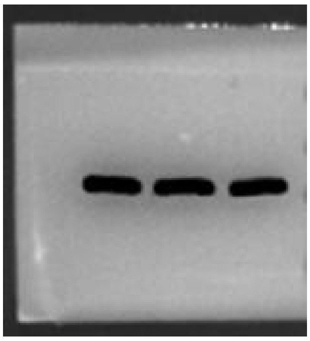

Figure 8. ACT1

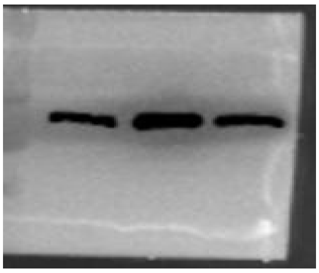

Figure 8. TRAF6

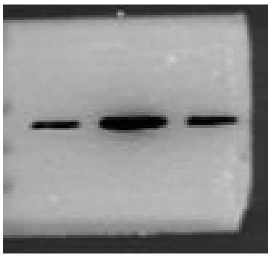

Figure 8. p-ERK

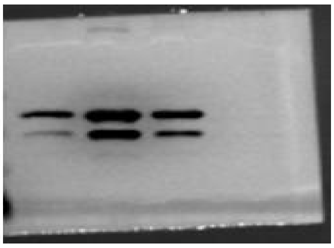

Figure 8. ERK

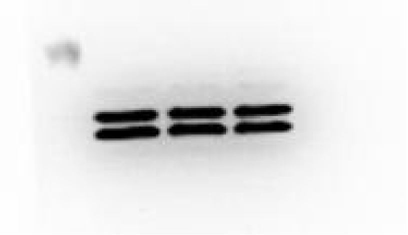

Figure 8. p-IKBα

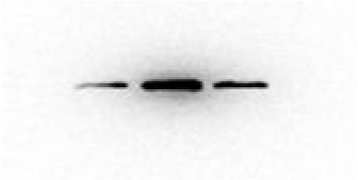

Figure 8. p-IKBα

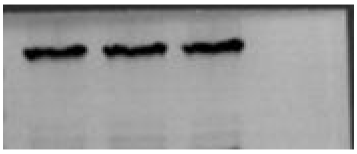

Figure 8. p-NF-κB

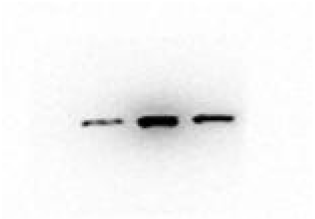

Figure 8.NF-κB

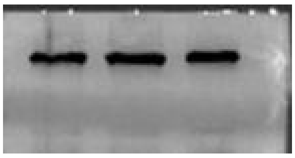

Figure 8. p-IKKβ

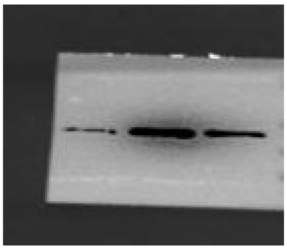

Figure 8.IKKβ

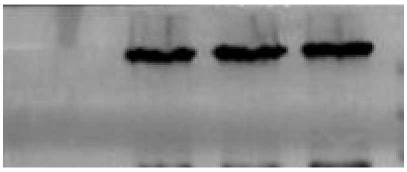

Figure 8. p-p38

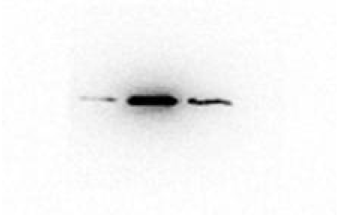

Figure 8. p38

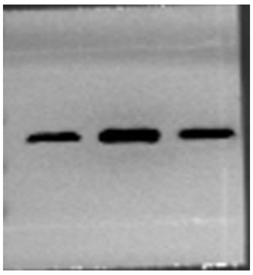

Figure 8. ZO-1

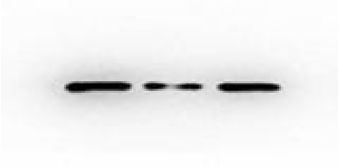

Figure 8. Occludin

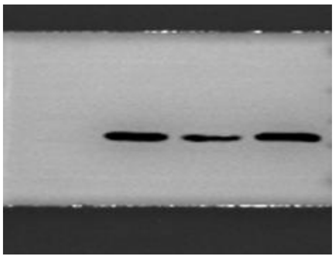

Figure 8. β-actin

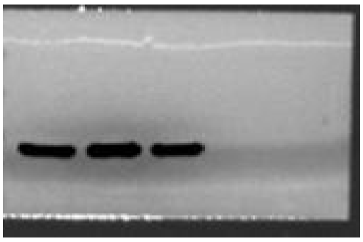

Supplement: Supplementary file 1 — Additional file 1. Original Western blot images. [file 40104_2026_1401_MOESM1_ESM.pdf]
