# Supplementary material for: Baicalin alleviates mastitis in dairy cows by targeting IL-17RA to inhibit IL-17 signaling pathway activation
Source: J Anim Sci Biotechnol. 2026 Apr 16;17:69. doi: 10.1186/s40104-026-01401-2 (PMC13085363; doi:10.1186/s40104-026-01401-2)
Supplement: Supplementary file 2 — Additional file 2. Fig. S1. Analysis of ACT1, TRAF6, ERK, IKBα, NF-κB, IKKβ, p38, IL-6, IL-1β, TNFα, ZO-1 and Occludin mRNA expression levels. Fig. S2. Analysis of IL-6, IL-1β, TNFα, MMP9, ACT1, TRAF6, S100A9, ZO-1 and Occludin mRNA expression levels. Fig.e S3. MD analysis of baicalin binding to IL-17RA. Fig. S4. Analysis of IL-6, IL-1β, TNFα, IL-17RA, ACT1, TRAF6, S100A9, ZO-1 and Occludin mRNA expression levels. Fig. S5. ZO-1 and Occludin mRNA expression levels. Fig. S6. Analysis of ACT1, TRAF6, ERK, IKBα, NF-κB, IKKβ, p38, ZO-1 and Occludin mRNA expression levels. Table S1. Primers for RT-qPCR for mouse species. Table S2. Primers for RT-qPCR for bovine species. Table S3. Antibody information. Table S4. Mouse IL-17RA CDS sequence information. Table S5. SgRNA sequence information. Table S6. TCMBANK selection results. [file 40104_2026_1401_MOESM2_ESM.docx]

Supporting Information for

**Baicalin alleviates mastitis in dairy cows by targeting IL-17RA to inhibit IL-17 signaling pathway activation**

Rui Feng^1^, Hefei Huang^1^, Qian Ma^1^, Weilin Gao^1^, Xu Chen^1^, Xiaoxue Yan^1^, Fan Wang^1^, Qian Zhang^1^, Yu Cao^2^, Han Zhang^1^, Junyang Teng^1^, Xin Ran^1^, Yong Zhang^1^, Shoupeng Fu^2^, Jun Liu^1^* and Xu Liu^1^*

1 Key Laboratory of Animal Biotechnology of the Ministry of Agriculture, College of Veterinary Medicine, Northwest Agriculture & Forestry University, Yangling, Shaanxi, 712100, China.

2 State Key Laboratory for Diagnosis and Treatment of Severe Zoonotic Infectious Diseases, Key Laboratory for Zoonosis Research of the Ministry of Education, Institute of Zoonosis, College of Veterinary Medicine, Jilin University, Changchun 130062, China.

Rui Feng, Hefei Huang and Qian Ma contributed equally to this study.

**This word file includes:**

Figure S1 to Figure S6

Table S1 to Table S6


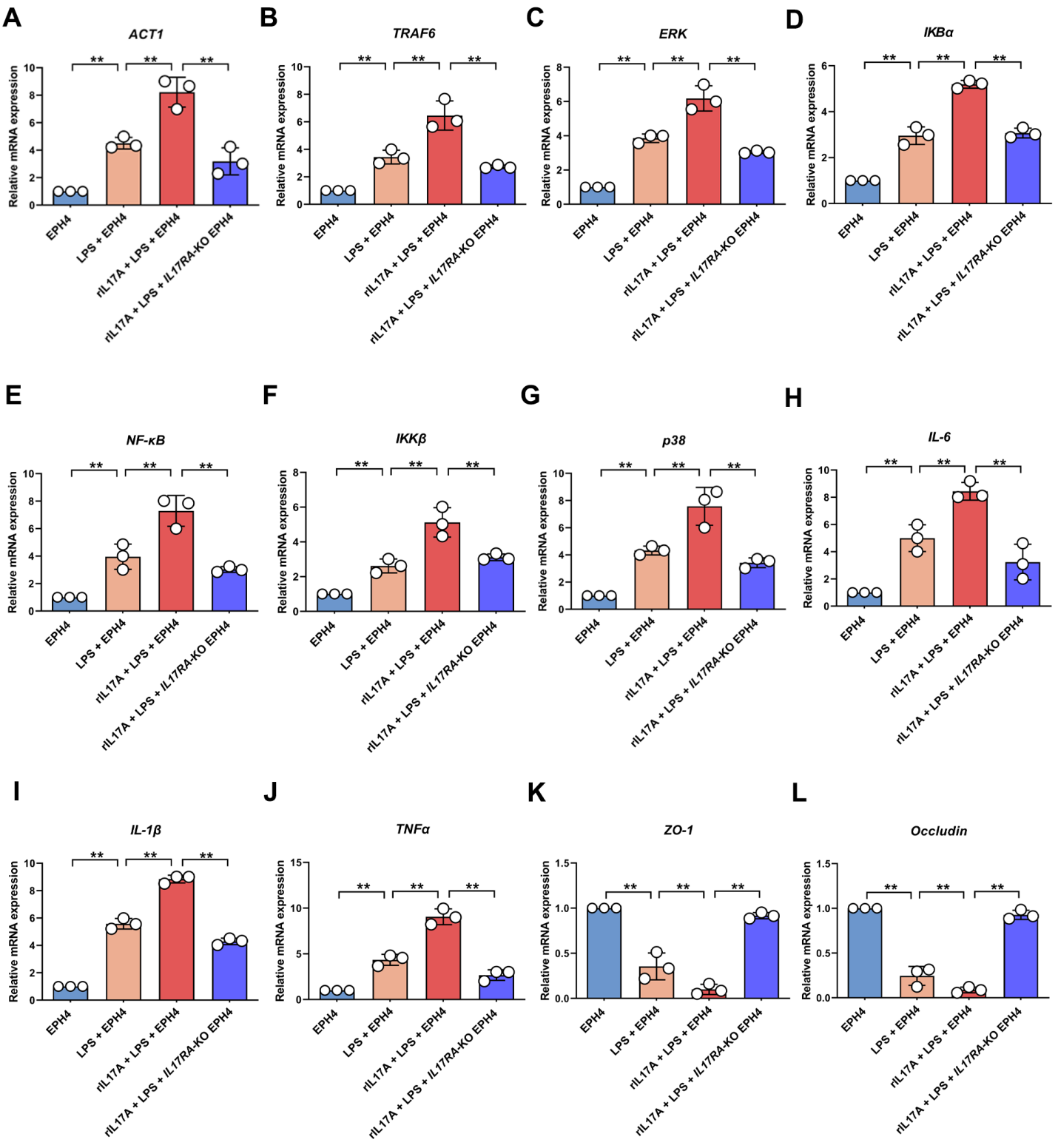
**Figure S1. Analysis of *ACT1*, *TRAF6*, *ERK*, *IKBα*, *NF-κB*, *IKKβ*, *p38*, *IL-6*, *IL-1β*, *TNFα*, *ZO-1* and *Occludin* mRNA expression levels.**

1. *ACT1*. (B) *TRAF6*. (C) *ERK*. (D) *IKBα*. (E) *NF-κB*. (F) *IKKβ*. (G) *p38*. (H) *IL-6*. (I) *IL-1β*. (J) *TNFα*. (K) *ZO-1*. (L) *Occludin*. Values are expressed as mean ± SEM (n = 3 per group), *: indicates significant difference (*P* < 0.05), **: indicates highly significant difference (*P* < 0.01).


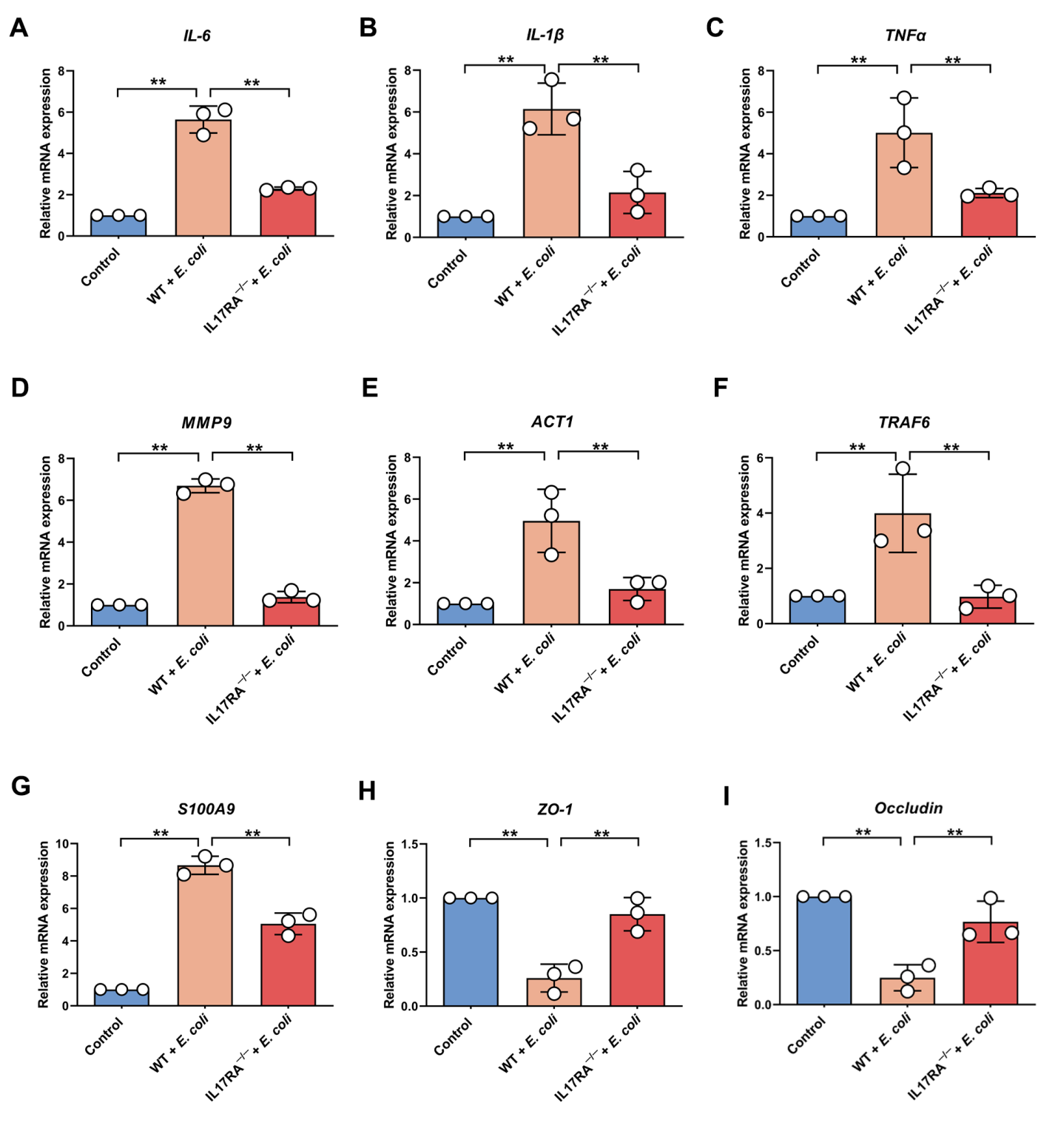
**Figure S2. Analysis of *IL-6*, *IL-1β*, *TNFα*, *MMP9*, *ACT1*, *TRAF6*, *S100A9*, *ZO-1* and *Occludin* mRNA expression levels.**

1. *IL-6*. (B) *IL-1β*. (C) *TNFα*. (D) *MMP9*. (E) *ACT1*. (F) *TRAF6*. (G) *S100A9*. (H) *ZO-1*. (I) *Occludin*. Values are expressed as mean ± SEM (n = 3 per group), *: indicates significant difference (*P* < 0.05), **: indicates highly significant difference (*P* < 0.01).


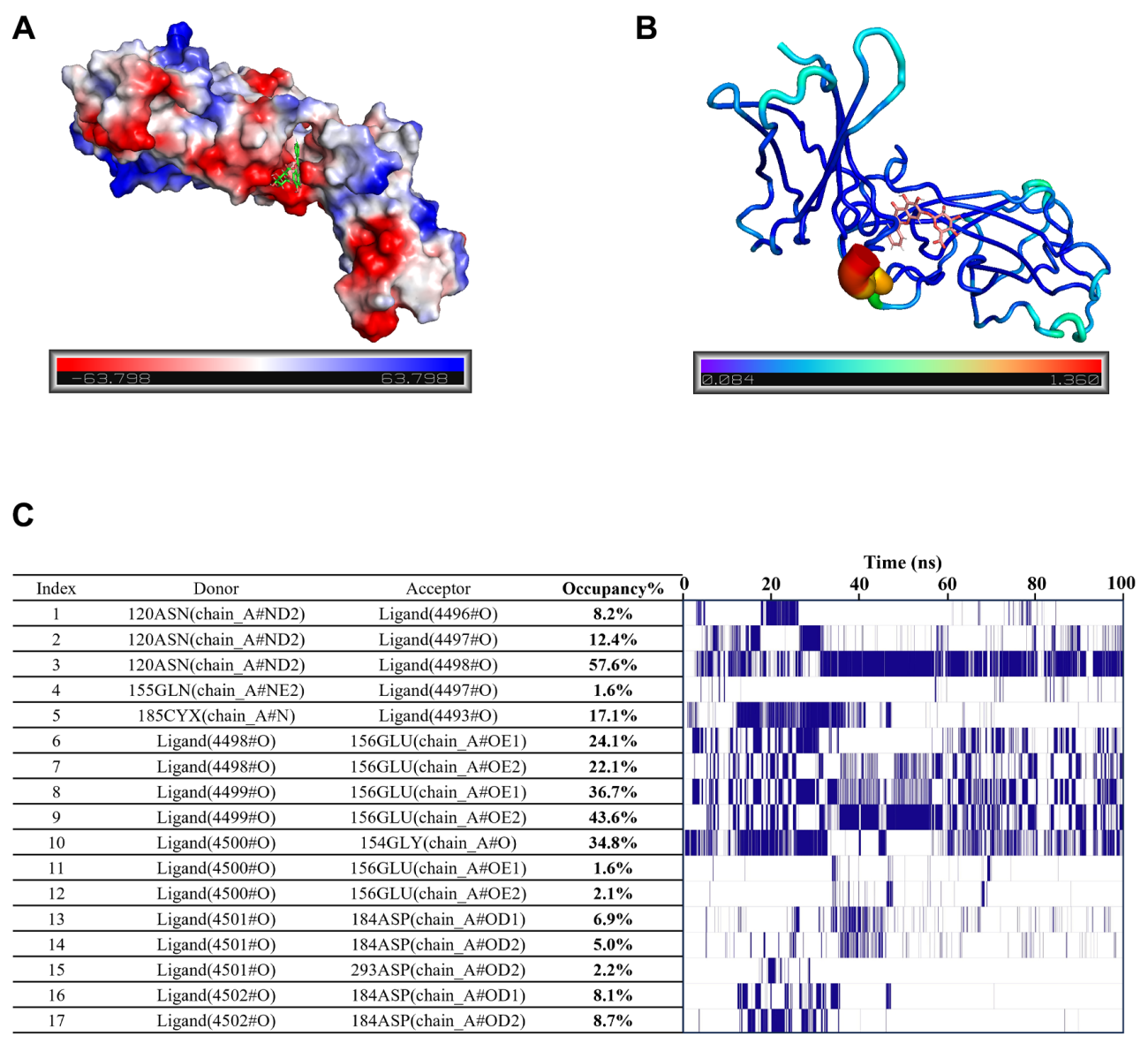
**Figure S3. MD analysis of baicalin binding to IL-17RA.**

(A) Surface electrostatic potential of small molecule-binding proteins (unit: kcal/mol). (B) B-factor plot constructed using root mean square fluctuation values as B-factors (unit: nm). (C) Hydrogen bond frequency between baicalin and IL-17RA proteins.

**
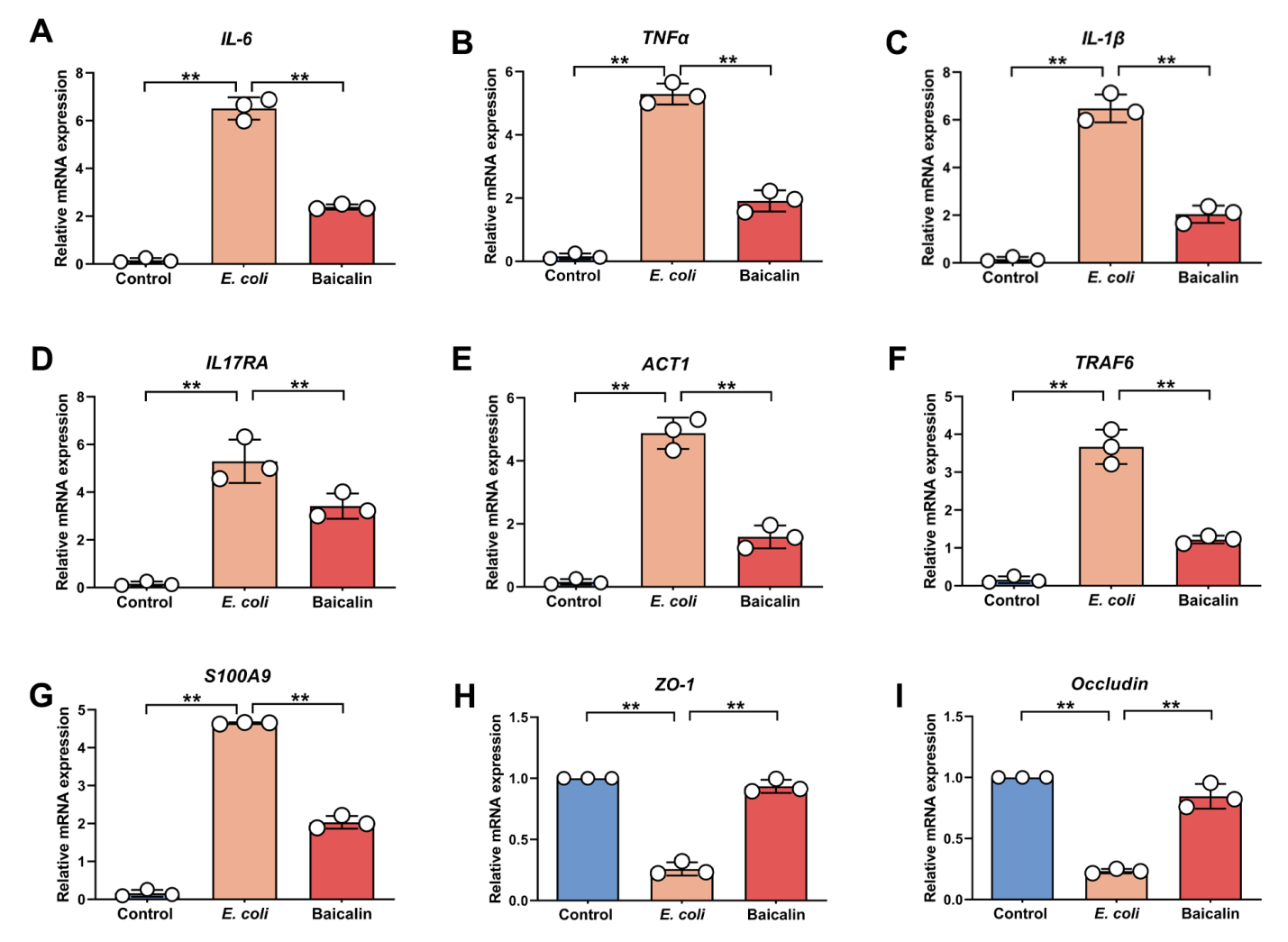
Figure S4. Analysis of *IL-6*, *IL-1β*, *TNFα*, *IL-17RA*, *ACT1*, *TRAF6*, *S100A9*, *ZO-1* and *Occludin* mRNA expression levels.**

1. *IL-6*. (B) *TNFα*. (C) *IL-1β*. (D) *IL-17RA*. (E) *ACT1*. (F) *TRAF6*. (G) *S100A9*. (H) *ZO-1*. (I) *Occludin*. Values are expressed as mean ± SEM (n = 3 per group), *: indicates significant difference (*P* < 0.05), **: indicates highly significant difference (*P* < 0.01).

**Figure S5. *ZO-1* and *Occludin* mRNA expression levels.**

1. *ZO-1*. (B) *Occludin*. Values are expressed as mean ± SEM (n = 3 per group), *: indicates significant difference (*P* < 0.05), **: indicates highly significant difference (*P* < 0.01).


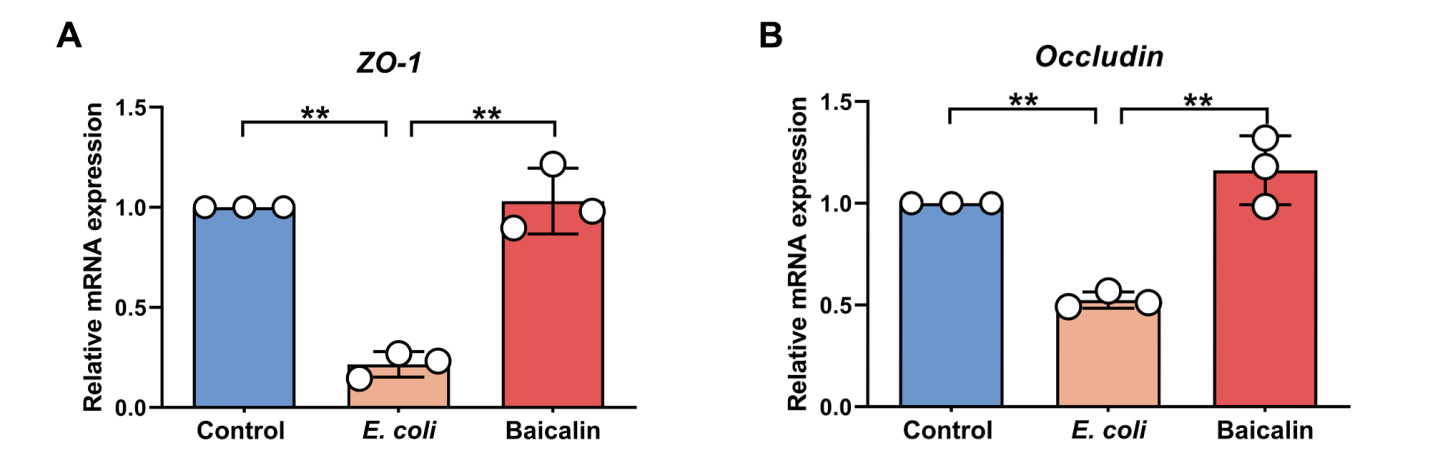


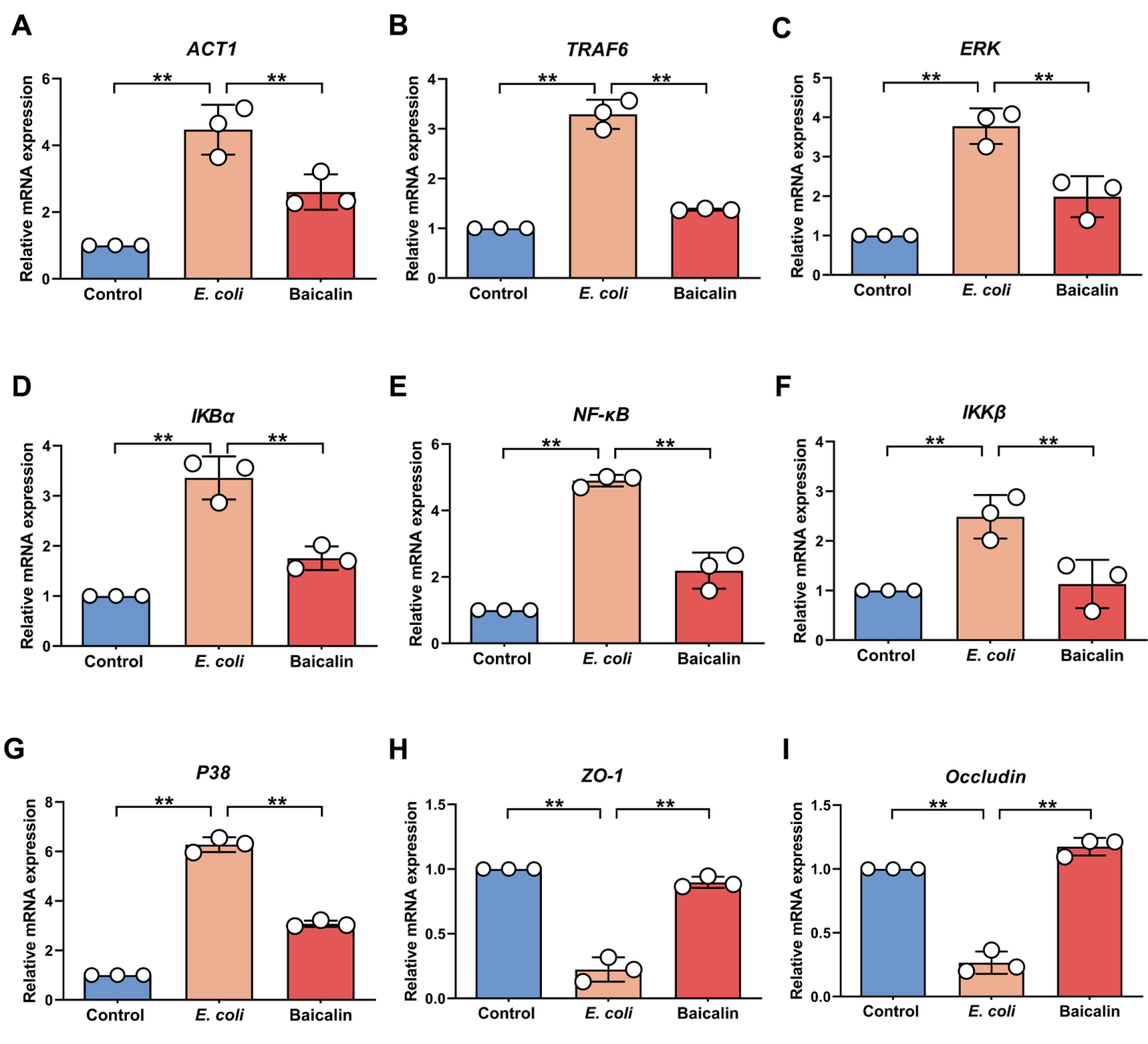
**Figure S6. Analysis of *ACT1*, *TRAF6*, *ERK*, *IKBα*, *NF-κB*, *IKKβ*, *p38*, *ZO-1* and *Occludin* mRNA expression levels.**

1. *ACT1*. (B) *TRAF6*. (C) *ERK*. (D) *IKBα*. (E) *NF-κB*. (F) *IKKβ*. (G) *p38*. (H) *ZO-1*. (I) *Occludin*. Values are expressed as mean ± SEM (n = 3 per group), *: indicates significant difference (*P* < 0.05), **: indicates highly significant difference (*P* < 0.01).

| **Table S1. Primers for RT-qPCR for mouse species** | | |
| --- | --- | --- |
| **Gene** | | **Primer sequence** |
| *IL-6*(NM_001314054.1) F | | GTTGCCTTCTTGGGACTGAT |
| *IL-6*(NM_001314054.1) R | CTGGCTTTGTCTTTCTTGTTAT | |
| *TNFα*(NM_001278601.1) F | GTGCCTATGTCTCAGCCTCTTC | |
| *TNFα*(NM_001278601.1) R | TCCTCCACTTGGTGGTTTGT | |
| *IL-1β*(NM_008361.4) F | AACTGCACTACAGGCTCCGAGA | |
| *IL-1β*(NM_008361.4) R | GCCACAGGTATTTTGTCGTTGCTT | |
| *ZO-1*(NM_001163574.2) F | GCCGCTAAGAGCACAGCAA | |
| *ZO-1*(NM_001163574.2) R | GCCCTCCTTTTAACACATCAGA | |
| *Occludin*(NM_001360536.1) F | | TTGAAAGTCCACCTCCTTACAGA |
| *Occludin*(NM_001360536.1) R | | CCGGATAAAAAGAGTACGCTGG |
| *β-actin*(NM_007393.5) F | | TGCTGTCCCTGTATGCCTCT |
| *β-actin*(NM_007393.5) R | | TTTGATGTCACGCACGATTT |
| *MMP9*(NM_013599.5)F | | TACGGACCCGAAGCGGACAT |
| *MMP9*(NM_013599.5)R | | CGCACCAGCGGTAACCATCC |
| *IL-17RA*(NM_008359.2)F | | TGCTGGAAAGTTTCTCCGACTC |
| *IL-17RA*(NM_008359.2)R | | GGCTTGGGAACTGTGGTATTT |
| *S100A9*(NM_001281852.1)F | | ATGGCCAACAAAGCACCTTCTCAGA |
| *S100A9*(NM_001281852.1)R | | TCTGAGAAGGTGCTTTGTTGGCCAT |
| *TRAF6*(NM_001303273.1)F | | AGTATGAGTGTCCCATCTGCT |
| *TRAF6*(NM_001303273.1)R | | TTTACCGTCAGGGAAAGAAT |
| *ERK*(NM_001038663.1)F | | ACCGTGACCTCAAGCCTTCC |
| *ERK*(NM_001038663.1)R | | GAGCCTGTTCAACTTCAATCCTCT |
| *IKBα*(NM_010907.2)F | | GAAGGACGAGGAGTACGAGCAA |
| *IKBα*(NM_010907.2)R | | GCCAGGTAGCCGTGGATAGAG |
| *NF-κB*(NM_001365067.1)F | | TGATGTGCATCGGCAAGTGG |
| *NF-κB*(NM_001365067.1)R | | GAAGTTGAGTTTCGGGTAGGC |
| *IKKβ*(NM_001159774.2)F | | TGACATCGCATCGGCTCTTA |
| *IKKβ*(NM_001159774.2)R | | ACTGCACAGGCTGCCAGTTA |
| *p38*(NM_001168508.1)F | | ACCTAAAGCCCAGCAACCTA |
| *p38*(NM_001168508.1)R | | AAACGAGCATCTTCTCCAGTA |

| **Table S2. Primers for RT-qPCR for bovine species** | |
| --- | --- |
| **Gene** | **Primer sequence** |
| *IKBα*(NM_001045868.1 )F | CGAGGAGTATGAGCAGATGGTG |
| *IKBα*(NM_001045868.1 )R | GCGATTTCTGGCTGGTTAGTG |
| *IL-17RA*(XM_015460734.3)F | GAGGCTACCAGGGTTCCGTCAA |
| *IL-17RA*(XM_015460734.3)R | AGCAGGTCCAGGGCTACTTCG |
| *S100A9*(NM_001046328.2)F | TGACACCCTGATCCAGAAAGA |
| *S100A9*(NM_001046328.2)R | GCCACCAGCATAATGAACTCC |
| ACT1(NM_001035483.1)F | GCAGCCTACCAGCAAGTGAT |
| *ACT1*(NM_001035483.1)R | TGGAAGCCGTTTACCAACAA |
| *TRAF6(NM_001034661.2)*F | GAACAGATGCCCAATCACTAT |
| *TRAF6(NM_001034661.2)*R | TACAAGGCGACCCTCTAACT |
| *IL-6*(NM_173923.2)F | TGTGAAAGCAGCAAGGAGACA |
| *IL-6*(NM_173923.2)R | CATCCGTCCTTTTCCTCCATT |
| *TNFα*(NM_173966.3)F | ACGGGCTTTACCTCATCTACTCA |
| *TNFα*(NM_173966.3)R | GGCTCTTGATGGCAGACAGG |
| *IL-1β*(NM_174093.1)F | GGCAACCGTACCTGAACCCA |
| *IL-1β*(NM_174093.1)R | CCACGATGACCGACACCACC |
| *β-actin*(NM_173979.3)F | GTCCGTGACATCAAGGAGAAGC |
| *β-actin*(NM_173979.3)R | AGCACCGTGTTGGCGTAGAG |
| *ERK(NM_175793.2)*F | ACCGTGACCTCAAACCTTCC |
| *ERK(NM_175793.2)*R | TGCTCCACCTCGATCCTCTT |
| *NF-κB(NM_001080242.2)*F | GAGCACGGACACCACCAAGA |
| *NF-κB(NM_001080242.2)*R | GCTCCCAGAGTTCCGATTCAC |
| *IKKβ(NM_174353.2)*F | CCTCCTGAAGATTGCGTGTAGC |
| *IKKβ(NM_174353.2)*R | GTTCCTCCTCCTCCGACTGC |
| *p38(NM_001102174.1)*F | ACGATCCTGACGACGAACCA |
| *p38(NM_001102174.1)*R | GCGGCACAAAGCTGATGACT |

**Table S3. Antibody information**

| **Protein name** | **Company** | **Catalogue no** | **Dilution rate** | **WB/IF** |
| --- | --- | --- | --- | --- |
| ACT1 | Bioss | bs-6202R | 1:500 | WB |
| TRAF6 | Bioss | bs-2830R | 1:500 | WB |
| IL-17RA | Bioss | bs-2606R | 1:500 | WB |
| MMP9 | Bioss | bsm-54040R | 1:500 | WB |
| S100A9 | Proteintech | 26992-1-AP | 1:500 | WB |
| Phospho-p38 | Proteintech | 28796-1-AP | 1:500 | WB |
| p38 | Proteintech | 14064-1-AP | 1:500 | WB |
| Phospho-IKKβ | Beyotime | AF5839 | 1:500 | WB |
| IKKβ | Beyotime | AI137 | 1:500 | WB |
| Phospho-ERK | Proteintech | 28733-1-AP | 1:500 | WB |
| ERK | Proteintech | 11257-1-AP | 1:500 | WB |
| Phospho-IκBα | Proteintech | 82349-1-RR | 1:500 | WB |
| IκBα | Proteintech | 10268-1-AP | 1:500 | WB |
| Phospho-NF-κB P65 | Proteintech | 3033 | 1:500 | WB |
| NF-κB P65 | Proteintech | 10745-1-AP | 1:100 | WB |
| ZO-1 | Proteintech | 21773-1-AP | 1:500 | WB/IF |
| Occludin | Proteintech | 66378-1-Ig | 1:500 | WB/IF |
| IL-6 | Proteintech | 66146-1-Ig | 1:500 | WB |
| TNFα | Proteintech | 60291-1-Ig | 1:500 | WB |
| IL-1β | Proteintech | 16806-1-AP | 1:500 | WB |
| β-actin | Proteintech | 20536-1-AP | 1:500 | WB |
| HRP-conjugated Affinipure GoatAnti-Mouse IgG(H+L) | Proteintech | SA00001-1 | 1:5000 | WB |
| HRP-conjugated Affinipure Goat Anti-Rabbit IgG(H+L) | Proteintech | SA00001-2 | 1:5000 | WB |
| Alexa Fluor 488 | Beyotime | A0428 | 1:500 | IF |
| Alexa Fluor 488 | Beyotime | A0423 | 1:500 | IF |

**Table S4. Mouse IL-17RA CDS sequence information**

| **Sequence Information** |
| --- |
| ATGGCGATTCGGCGCTGCTGGCCACGGGTCGTCCCCGGGCCCGCGCTGGGATGGCTGCTTCTGCTGCTGAACGTTCTGGCCCCGGGCCGCGCCTCCCCGCGCCTCCTCGACTTCCCGGCTCCGGTCTGCGCGCAGGAGGGGCTGAGCTGCAGAGTCAAGAATAGTACTTGTCTGGATGACAGCTGGATCCACCCCAAAAACCTGACCCCGTCTTCCCCAAAAAACATCTATATCAATCTTAGTGTTTCCTCTACCCAGCACGGAGAATTAGTCCCTGTGTTGCATGTTGAGTGGACCCTGCAGACAGATGCCAGCATCCTGTACCTCGAGGGTGCAGAGCTGTCCGTCCTGCAGCTGAACACCAATGAGCGGCTGTGTGTCAAGTTCCAGTTTCTGTCCATGCTGCAGCATCACCGTAAGCGGTGGCGGTTTTCCTTCAGCCACTTTGTGGTAGATCCTGGCCAGGAGTATGAAGTGACTGTTCACCACCTGCCGAAGCCCATCCCTGATGGGGACCCAAACCACAAATCCAAGATCATCTTTGTGCCTGACTGTGAGGACAGCAAGATGAAGATGACTACCTCATGCGTGAGCTCAGGCAGCCTTTGGGATCCCAACATCACTGTGGAGACCTTGGACACACAGCATCTGCGAGTGGACTTCACCCTGTGGAATGAATCCACCCCCTACCAGGTCCTGCTGGAAAGTTTCTCCGACTCAGAGAACCACAGCTGCTTTGATGTCGTTAAACAAATATTTGCGCCCAGGCAAGAAGAATTCCATCAGCGAGCTAATGTCACATTCACTCTAAGCAAGTTTCACTGGTGCTGCCATCACCACGTGCAGGTCCAGCCCTTCTTCAGCAGCTGCCTAAATGACTGTTTGAGACACGCTGTGACTGTGCCCTGCCCAGTAATCTCAAATACCACAGTTCCCAAGCCAGTTGCAGACTACATTCCCCTGTGGGTGTATGGCCTCATCACACTCATCGCCATTCTGCTGGTGGGATCTGTCATCGTGCTGATCATCTGTATGACCTGGAGGCTTTCTGGCGCCGATCAAGAGAAACATGGTGATGACTCCAAAATCAATGGCATCTTGCCCGTAGCAGACCTGACTCCCCCACCCCTGAGGCCCAGGAAGGTCTGGATCGTCTACTCGGCCGACCACCCCCTCTATGTGGAGGTGGTCCTAAAGTTCGCCCAGTTCCTGATCACTGCCTGTGGCACTGAAGTAGCCCTTGACCTCCTGGAAGAGCAGGTTATCTCTGAGGTGGGGGTCATGACCTGGGTGAGCCGACAGAAGCAGGAGATGGTGGAGAGCAACTCCAAAATCATCATCCTGTGTTCCCGAGGCACCCAAGCAAAGTGGAAAGCTATCTTGGGTTGGGCTGAGCCTGCTGTCCAGCTACGGTGTGACCACTGGAAGCCTGCTGGGGACCTTTTCACTGCAGCCATGAACATGATCCTGCCAGACTTCAAGAGGCCAGCCTGCTTCGGCACCTACGTTGTTTGCTACTTCAGTGGCATCTGTAGTGAGAGGGATGTCCCCGACCTCTTCAACATCACCTCCAGGTACCCACTCATGGACAGATTTGAGGAGGTTTACTTCCGGATCCAGGACCTGGAGATGTTTGAACCCGGCCGGATGCACCATGTCAGAGAGCTCACAGGGGACAATTACCTGCAGAGCCCTAGTGGCCGGCAGCTCAAGGAGGCTGTGCTTAGGTTCCAGGAGTGGCAAACCCAGTGCCCCGACTGGTTCGAGCGTGAGAACCTCTGCTTAGCTGATGGCCAAGATCTTCCCTCCCTGGATGAAGAAGTGTTTGAAGACCCACTGCTGCCACCAGGGGGAGGAATTGTCAAACAGCAGCCCCTGGTGCGGGAACTCCCATCTGACGGCTGCCTTGTGGTAGATGTCTGTGTCAGTGAGGAAGAAAGTAGAATGGCAAAGCTGGACCCTCAGCTATGGCCACAGAGAGAGCTAGTGGCTCACACCCTCCAAAGCATGGTGCTGCCAGCAGAGCAGGTCCCTGCAGCTCATGTGGTGGAGCCTCTCCATCTCCCAGACGGCAGTGGAGCAGCTGCCCAGCTGCCCATGACAGAGGACAGCGAGGCTTGCCCGCTGCTGGGGGTCCAGAGGAACAGCATCCTTTGCCTCCCCGTGGACTCAGATGACTTGCCACTCTGTAGCACCCCAATGATGTCACCTGACCACCTCCAAGGCGATGCAAGAGAGCAGCTAGAAAGCCTAATGCTCTCGGTGCTGCAGCAGAGCCTGAGTGGACAGCCCCTGGAGAGCTGGCCGAGGCCAGAGGTGGTCCTCGAGGGCTGCACACCCTCTGAGGAGGAGCAGCGGCAGTCGGTGCAGTCGGACCAGGGCTACATCTCCAGGAGCTCCCCGCAGCCCCCCGAGTGGCTCACGGAGGAGGAAGAGCTAGAACTGGGTGAGCCCGTTGAGTCTCTCTCTCCTGAGGAACTACGGAGCCTGAGGAAGCTCCAGAGGCAGCTTTTCTTCTGGGAGCTCGAGAAGAACCCTGGCTGGAACAGCTTGGAGCCACGGAGACCCACCCCAGAAGAGCAGAATCCCTCCTAG |

**Table S5. SgRNA sequence information**

| **SgRNA name** | **Sequence information** |
| --- | --- |
| Cas9 sgRNA1 | GCTCGAACCAGTCGGGGCACTGG |
| Cas9 sgRNA2 | TCTCACGCTCGAACCAGTCGGGG |
| Cas9 sgRNA3 | GTTCTCACGCTCGAACCAGTCGG |
| Cas9 sgRNA4 | TCCAGTGGTCACACCGTAGC TGG |
| Cas9 sgRNA5 | GGAGGTTTACTTCCGGATCC AGG |
| Cas9 sgRNA6 | AACAACGTAGGTGCCGAAGCAGG |

**Table S6. TCMBANK selection results**

| \| **TCM BANK ID** \| \| --- \| | **Detailed information** | **Small molecule name** |
| --- | --- | --- | --- |
| TCMBANKIN054427 | https://tcmbank.cn/Ingredients/detail?ID=TCMBANKIN054427 | Baicalin |
